# Pathobionts in the Vaginal Microbiota: Individual Participant Data Meta-Analysis of Three Sequencing Studies

**DOI:** 10.3389/fcimb.2020.00129

**Published:** 2020-04-15

**Authors:** Janneke H. H. M. van de Wijgert, Marijn C. Verwijs, A. Christina Gill, Hanneke Borgdorff, Charlotte van der Veer, Philippe Mayaud

**Affiliations:** ^1^Department of Clinical Infection, Microbiology, and Immunology, Institute of Infection and Global Health, University of Liverpool, Liverpool, United Kingdom; ^2^Julius Center for Health Sciences and Primary Care, University Medical Center Utrecht, Utrecht University, Utrecht, Netherlands; ^3^Amsterdam Institute for Global Health and Development, Amsterdam UMC, Amsterdam, Netherlands; ^4^Amsterdam Public Health Service, Amsterdam, Netherlands; ^5^London School of Hygiene and Tropical Medicine, London, United Kingdom

**Keywords:** vaginal microbiota, bacterial vaginosis, *Streptococcus*, *Staphylococcus*, *Enterococcus*, *Escherichia*, pathobionts, ethnicity

## Abstract

Sequencing studies have shown that optimal vaginal microbiota (VMB) are lactobacilli-dominated and that anaerobes associated with bacterial vaginosis (BV-anaerobes) are commonly present. However, they overlooked a less prevalent but more pathogenic group of vaginal bacteria: the pathobionts that cause maternal and neonatal infections and pelvic inflammatory disease. We conducted an individual participant data meta-analysis of three VMB sequencing studies that included diverse groups of women in Rwanda, South Africa, and the Netherlands (2,044 samples from 1,163 women in total). We identified 40 pathobiont taxa but only six were non-minority taxa (at least 1% relative abundance in at least one sample) in all studies: *Streptococcus* (54% of pathobionts reads)*, Staphylococcus, Enterococcus, Escherichia/Shigella, Haemophilus*, and *Campylobacter*. When all pathobionts were combined into one bacterial group, the VMB of 17% of women contained a relative abundance of at least 1%. We found a significant negative correlation between relative abundances (ρ = −0.9234), but not estimated concentrations (*r* = 0.0031), of lactobacilli and BV-anaerobes; and a significant positive correlation between estimated concentrations of pathobionts and BV-anaerobes (*r* = 0.1938) but not between pathobionts and lactobacilli (*r* = 0.0436; although lactobacilli declined non-significantly with increasing pathobionts proportions). VMB sequencing data were also classified into mutually exclusive VMB types. The overall mean bacterial load of the ≥20% pathobionts VMB type (5.85 log_10_ cells/μl) was similar to those of the three lactobacilli-dominated VMB types (means 5.13–5.83 log_10_ cells/μl) but lower than those of the four anaerobic dysbiosis VMB types (means 6.11–6.87 log_10_ cells/μl). These results suggest that pathobionts co-occur with both lactobacilli and BV-anaerobes and do not expand as much as BV-anaerobes do in a dysbiotic situation. Pathobionts detection/levels were increased in samples with a Nugent score of 4–6 in both studies that conducted Nugent-scoring. Having pathobionts was positively associated with young age, non-Dutch origin, hormonal contraceptive use, smoking, antibiotic use in the 14 days prior to sampling, HIV status, and the presence of sexually transmitted pathogens, in at least one but not all studies; inconsistently associated with sexual risk-taking and unusual vaginal discharge reporting; and not associated with vaginal yeasts detection by microscopy. We recommend that future VMB studies quantify common vaginal pathobiont genera.

## Introduction

Understanding of the vaginal microbiota (VMB) has increased significantly since the turn of the century due to the increased availability of molecular laboratory techniques such as next-generation sequencing (van de Wijgert et al., 2014). Molecular studies have shown that most women have a VMB consisting of lactobacilli (most commonly *Lactobacillus crispatus* or *L. iners*), but that vaginal dysbiosis is highly prevalent worldwide (van de Wijgert and Jespers, 2017). The most common type of vaginal dysbiosis is anaerobic dysbiosis, which is characterized by a decrease of lactobacilli and an increase of fastidious anaerobes (van de Wijgert et al., 2014). Clinicians refer to symptomatic anaerobic dysbiosis as bacterial vaginosis (BV): patients typically have mild vaginal inflammation and a fishy-smelling vaginal discharge. It should be noted, however, that anaerobic dysbiosis is also frequently asymptomatic. The VMB of most women with anaerobic dysbiosis consists of a highly diverse mixture of fastidious anaerobes, usually including *Gardnerella vaginalis*. However, a substantial proportion of women with anaerobic dysbiosis are dominated by *G. vaginalis*, and this type of low diverse anaerobic dysbiosis is often overlooked. Recent studies have suggested that these women might be more difficult to treat, potentially due to the presence of a *G. vaginalis*-initiated vaginal mucosal biofilm (Verwijs et al., 2019a).

Another clinically relevant type of vaginal dysbiosis that has systematically been overlooked is the presence of bacterial pathobionts in the VMB (van de Wijgert and Jespers, 2017). Microbiologists define the term pathobiont as any potentially pathological organism which, under normal circumstances, lives as a non-harming symbiont. In the vaginal niche, this would include—among others—*Streptococcus agalactiae* (Group B streptococcus), *Staphylococcus aureus*, and species in the *Enterobacteriaceae* family. These bacteria have often been associated with maternal and neonatal infections (Cools et al., 2016; Black et al., 2018), as well as invasive infections in non-pregnant women such as pelvic inflammatory disease (Brunham et al., 2015). Some clinical researchers have hypothesized that a distinct type of vaginitis (desquamative inflammatory vaginitis), which is characterized by much more severe vaginal inflammation than BV and with desquamation of vaginal epithelial cells including parabasal cells (Sobel, 1994; Paavonen and Brunham, 2018), may be caused by pathobionts in the VMB (Donders et al., 2017). Two cases that appear to have been triggered by toxic shock syndrome toxin-1-producing *Staphylococcus aureus* strains have indeed been reported (Pereira et al., 2013). However, others believe that the condition is caused by estrogen deficiency or an immunologic disorder, and that vaginal dysbiosis develops secondarily (Sobel et al., 2011). A recent study found that most patients with vaginitis, parabasal cells, and lactobacilli-deficiency by microscopy did not have consistent VMB patterns by VMB sequencing (Oerlemans, 2019). We conclude that there is sufficient evidence to consider VMB pathobionts clinically relevant, but that the evidence-base related to both symptoms and complications is weak.

An important reason why the evidence-base is weak is because pathobionts are often not assessed properly. For example, neonatal invasive infection studies have focused on only one pathobiont (*S. agalactiae*) by culture (Kwatra et al., 2016), and VMB sequencing studies have systematically under-reported pathobionts. Authors of such studies typically use bioinformatical methods, such as hierarchical clustering, to summarize the sequencing data into a few VMB types. The first set of VMB types were published by Ravel et al. (2011) based on a study in asymptomatic American women: they referred to these as community state types I (*L. crispatus*-dominated), II (*L. gasseri*-dominated), III (*L. iners*-dominated), IV (diverse group), and V (*L. jensenii*-dominated (Ravel et al., 2011). The only pathobiont that was mentioned in this publication was *Streptococcus*, as one of the taxa included in the “diverse group.” However, hierarchical clustering only takes relative abundances into account and not the pathogenic potential of individual bacteria. Pathobionts usually occur at lower levels than BV-anaerobes, but have a higher pathogenic potential: these lower levels may therefore be clinically relevant. Because pathobionts rarely dominate the VMB, samples that contain pathobionts are often classified based on the other bacteria that are also present in that sample. For example, a sample containing 70% *L. iners* and 30% *S. agalactiae* would be classified as community state type III (*L. iners*-dominated) in most studies.

We believe that this vaginal pathobionts knowledge gap is hampering clinical progress in the field. We therefore performed an individual participant data meta-analysis of three VMB sequencing studies that enrolled diverse groups of women in Rwanda, South Africa, and the Netherlands (2,044 samples from 1,163 women in total), with as main aim to describe the presence and levels of all pathobionts identified in the sequencing data, their correlations with lactobacilli and BV-anaerobes, and their associations with participant sociodemographic, behavioral, and clinical/laboratory characteristics.

## Materials and Methods

### Studies Included in the Meta-Analysis

We performed an individual participant data meta-analysis of three VMB sequencing studies that were conducted in three different countries to account for regional and ethnic differences in VMB composition: (1) a clinical trial of intermittent oral metronidazole or vaginal probiotic use in Kigali, Rwanda (referred to as the Rwanda VMB study); (2) the VMB sub-study of the South African HPV in Africa Research Partnership (HARP) study in Johannesburg, South Africa; and (3) the VMB sub-study of the Healthy Life in an Urban Setting (HELIUS) study in Amsterdam, the Netherlands.

The Rwanda VMB study screened HIV-negative, non-pregnant, pre-menopausal women at high risk of sexually transmitted infections (STIs) for BV (van de Wijgert et al., 2020a). Women with BV were treated with metronidazole for seven days, and when cured of BV and other urogenital infections, were randomized to no intervention, or intermittent use of oral metronidazole or two different lactobacilli-containing vaginal probiotics for 2 months. The lactobacilli contained in the vaginal probiotics did not include any naturally occurring vaginal lactobacilli. Women were sampled at screening (start of BV/urogenital infection treatment, if applicable), enrollment (start of the interventions), Day 7, Month 1, Month 2 (cessation of the interventions), and Month 6. The study found that all three interventions were safe and affected naturally occurring lactobacilli and BV-anaerobes (in favor of the lactobacilli, particularly *L. iners*), but not pathobionts. However, to avoid any bias in this meta-analysis due to exposure to interventions, we conducted analyses that included lactobacilli and BV-anaerobes levels on VMB data that were not influenced by the interventions (*N* = 366 of 629 samples): data from samples collected in all randomization groups at the screening visit prior to any treatments (if applicable) and at the Month 6 visit (4 months after cessation of the interventions), as well as samples collected in the no intervention group at the Month 1 and Month 2 visits.

The VMB sub-study of the HARP study was a nested case-control study within a prospective cohort study conducted in Johannesburg, South Africa (van de Wijgert et al., 2020b). The study enrolled HIV-positive women and investigated the associations of VMB composition with high-risk human papillomavirus (hrHPV) and cervical intraepithelial neoplasia (CIN) acquisition, clearance, and/or persistence. All but one participant were of sub-Saharan African origin. Samples for VMB analyses were collected at baseline (*N* = 445) and at endline (*N* = 414), a median of 16 months later. The study concluded that hrHPV infection (and/or increased sexual risk-taking) likely causes anaerobic vaginal dysbiosis, but that a bidirectional relationship is also possible. Furthermore, in this population, dysbiosis did not increase CIN2+ risk, but CIN2+ increased dysbiosis risk. Since the study did not include an intervention, we used all available VMB data for the analyses presented in this paper.

The HELIUS study is a large, multi-ethnic cohort study in Amsterdam, the Netherlands (Snijder et al., 2017). Sampling was stratified by ethnic group and included the six largest ethnic groups in the city (Dutch, African Surinamese, South-Asian Surinamese, Turkish, Moroccan, and Ghanaian). In a sub-sample, a cross-sectional study on the association of ethnicity with VMB composition was performed (Borgdorff et al., 2017). For this sub-study, vaginal samples of 546 pre-menopausal women were sequenced. The most prevalent VMB composition in ethnically Dutch women was a *L. crispatus*-dominated VMB, in African Surinamese and Ghanaian women a polybacterial *G. vaginalis*-containing VMB, and in the other ethnic groups a *L. iners*-dominated VMB. This study did not include an intervention either, and we therefore used all available VMB data for the analyses presented in the current paper.

### Sequencing and Other Laboratory Methods

All three studies extracted DNA from vaginal swabs and conducted 16S rRNA gene sequencing of the V3–V4 variable regions on Illumina platforms (San Diego, CA, USA) as described by Fadrosh et al. (2014). Standard diagnostic tests were used to test for STIs (all three studies), and BV and vulvovaginal candidiasis (Rwanda VMB and HARP studies only). There were some differences in sequencing and diagnostic methods used, as outlined in Table 1. Because of these differences, we conducted all analyses on each study separately as well as the three studies combined.

**Table 1:** Main study characteristics of the three studies.

	**VMB Rwanda^a^**	**HARP South Africa^a^**	**HELIUS Netherlands^a^**
Number of women	162 (68 randomized)	455	546
Number of baseline samples	162	445	546
Total number of samples	629 (366 not influenced by interventions)	869	546
Years samples collected	2015–2016	2011–2014	2011–2013
Year samples sequenced	2017	2016	2014
Study location	Kigali	Johannesburg	Amsterdam
Study population	HIV-negative women with high sexual risk	HIV-positive women on cART (2/3) or not on cART (1/3)	Random samples of city population, stratified by ethnic group
**Sequencing methods**			
Sequencing laboratory	University of Liverpool, Center for Genomic Research	University of Liverpool, Center for Genomic Research	Amsterdam University Medical Center, location VUmc
Type of vaginal samples	Vaginal swab frozen dry the same day at −80°C and shipped to Liverpool on dry ice.	Vaginal swab in Boonfix, stored and shipped to Liverpool at room temperature.	Vaginal swab frozen dry at −20°C after at most 6 days at 2–8°C.
DNA extraction method	Lysozyme lysis with bead-beating, followed by Qiagen DNeasy Blood and Tissue kit	Lysozyme lysis (no bead-beating) followed by Qiagen DNeasy Blood and Tissue kit	Lysozyme, mutanolysin, lysostaphin lysis with bead-beating followed by proteinase K/RNase A and ChemaGen extraction robot
16S sequencing platform	Illumina HiSeq (rapid mode; 2 × 300bp)	Illumina HiSeq (rapid mode; 2 × 300bp)	Illumina MiSeq (2 × 300bp)
16S variable region	V3–V4	V3–V4	V3–V4
Taxonomic assignment and unit of analysis	DADA2 v1.4.0 ASV	Swarm v2.1.13 OTU	USEARCH v5.2.236 OTU
Reference databases	Silva v128, NCBI, Vaginal 16S rDNA Reference Database by Srinivasan et al.	Silva v128, NCBI	GreenGenes v13.8, NCBI, Vaginal 16S rDNA Reference Database by Srinivasan et al.
Rarefaction	At 1,111 reads	At 1,039 reads	None, but all samples with <100 reads discarded
Mean read count per sample	374,543	122,490	25,392
Unique ASVs/OTUs^b^	401 (177 non-minority)	1,981 (246 non-minority)	455 (141 non-minority)
BactQuant assay done^c^	Yes	No	No
**Diagnostic tests**			
Bacterial vaginosis	Nugent, Amsel	Nugent	Not done
Yeasts	Wet mount microscopy	Gram stain microscopy	Not done
*Trichomonas vaginalis*	Wet mount microscopy and InPouch culture	APTIMA Combo 2 PCR	APTIMA PCR
*Chlamydia trachomatis* and *Neisseria gonorrhoeae*	Presto or GeneXpert real-time PCR	APTIMA Combo 2 PCR	APTIMA Combo 2 PCR
Syphilis	Spinreact RPR + TPHA	Immutrep RPR + TPHA	Not done
HIV-1	National algorithm (serology)	National algorithm (serology)	Not done
Herpes simplex type 2	Kalon IgG2 ELISA	Kalon IgG2 ELISA	Not done
HPV	Not done	Digene HC-II, CareHPV, INNO-LiPA HPV Genotyping Extra	SPF10-PCR-DEIA/LiPA25 system version 1

*ASV, Amplicon Sequence Variant; cART, combination antiretroviral therapy for HIV; ELISA, enzyme-linked immunosorbent assay; HIV, human immunodeficiency virus; HPV, human papillomavirus; IgG, Immunoglobulin G; NCBI, National Center for Biotechnology Information; OTU, Operational Taxonomic Unit; PCR, polymerase chain reaction; RPR, Rapid Plasma Reagin test; TPHA, Treponema pallidum Hemagglutination Assay*.

a *Details of procedures and supplies/databases used can be found in the original publications (Borgdorff et al., 2017; van de Wijgert et al., 2020a,b)*.

b *Non-minority is defined as at least 1% in at least one sample. The number of minority OTUs was higher in the HARP study than in the other two studies because OTUs matching to the same or overlapping taxa were not merged. This has, however, not affected the analyses in this paper, which were based on bacterial groups and a select number of non-minority taxa*.

c *BactQuant is a commercial assay that quantifies 16S genes in a sample by quantitative PCR (Liu et al., 2012)*.

The BactQuant 16S rRNA gene qPCR (Liu et al., 2012) was only done in the Rwanda VMB study (*N* = 379, of which 158 samples were not influenced by the interventions). The 16S rRNA gene concentration per sample was used to convert the relative abundances of that sample into estimated concentrations as previously described (van de Wijgert et al., 2020a). We used both relative abundances as well as estimated concentrations for the Rwanda VMB study, but only had relative abundances for the other two studies.

### Sequencing Data Processing

The 16S rRNA gene sequencing and initial data processing yielded two-dimensional tables with samples and bacterial taxa on the axes, and relative abundances in the cells, for each of the three studies. DADA2 assigns amplicon sequence variants (ASVs) to taxa (in the Rwanda VMB study) and Swarm and USEARCH assign them to operational taxonomic units (OTUs; in the HARP and HELIUS studies, respectively). Details on quality control and cleaning of reads, taxonomic assignments, conversion of read counts into relative abundances, and rarefaction are summarized in Table 1 and explained in the original publications. The three study-specific relative abundance tables were combined into a single table, and all subsequent data processing steps were redone for the combined table (i.e., are slightly different from the original publications) to ensure that they were identical for the three studies.

Data reduction for biostatistical modeling was done in three different ways. First, the Simpson diversity index (1-D) was calculated for each sample, ranging from 0 (no diversity) to 1 (infinite diversity). Second, each ASV/OTU was assigned to one of four “bacterial groups” based on the published literature (Supplementary Material 1) as follows: (1) lactobacilli; (2) BV-anaerobes; (3) pathobionts; and (4) a rest group called “other bacteria” (which contained mostly skin and Bifidobacteria). Pathobionts were defined as all bacterial taxa that have been reported in the literature as having been associated with invasive disease, and are not typically associated with BV; we also included STI pathogens in this category because their mean relative abundances were too low to justify a separate bacterial group. For each sample, relative abundances of ASVs/OTUs belonging to the same bacterial group were summed. This resulted in four relative abundances (one for each bacterial group) per sample, which sum to one in total. For example, one sample could contain 0.5 (50%) lactobacilli reads, 0.4 (40%) BV-anaerobes reads, 0.08 (8%) pathobionts reads, and 0.02 (2%) other bacteria reads. Third, we classified samples into nine VMB types (with each sample assigned to only one VMB type): (1) *Lactobacillus iners*-dominated (Li; ≥75% relative abundance of lactobacilli of which *L. iners* was the most common); (2) *L. crispatus*-dominated (Lcr; also ≥75% lactobacilli of which *L. crispatus* was the most common); (3) dominated by other *Lactobacillus* species (Lo; also containing ≥75% lactobacilli); (4) lactobacilli and anaerobes (LA; ≥25% lactobacilli with the remainder BV-anaerobes); (5) high diversity BV-anaerobes with ≥10% *G. vaginalis* presence (BV_GV); (6) high diversity BV-anaerobes with <10% *G. vaginalis* presence (BV_noGV); (7) *G. vaginalis*-dominated (GV; *G. vaginalis* ≥50%); (8) substantial presence of pathobionts (PB; ≥20% pathobiont taxa); and (9) *Bifidobacterium*-dominated (BD; ≥50% Bifidobacteria).

### Statistical Analyses and Figures

Statistical analyses were performed using Stata version 13 (StataCorp, College Station, TX, USA) and R version 3.2.3 (R foundation for Statistical Computing 2016, Vienna, Austria). All analyses were cross-sectional, sometimes including samples collected at baseline only (one sample per woman) and sometimes including all samples (in case of the Rwanda VMB and HARP studies, more than one sample per woman). Women in the Rwanda VMB study were exposed to antibiotic and/or probiotic interventions, and samples that could potentially have been influenced by these interventions were excluded from most analyses as described above and as indicated in the tables and text. Unadjusted differences between groups of interest were tested by Fisher's exact test for binary variables, Chi-squared test for categorical variables, and Kruskal-Wallis test for continuous variables. Pathobiont levels (relative abundances or estimated concentrations) were correlated with those of other bacterial groups or taxa by Spearman's rank correlation when all samples were included and by Pearson's correlation coefficient when samples with <1% pathobionts were excluded. To assess sociodemographic, behavioral, and clinical determinants of pathobionts detection (≥1% compared to <1%) and levels, we used unadjusted logistic regression models for analyses including one sample per woman, and Kruskal-Wallis tests for analyses of pathobionts levels that included all samples. The heatmap was made with the *gplots* package in R (Warnes et al., 2016), bar charts in Stata, and correlation matrices with the *corrplot* package in R (Taiyun, 2019).

## Results

### Participant Characteristics

The median age in the three studies combined was 30 years (inter-quartile range 26–34) and most women were non-pregnant by design (Table 2). The majority of women in the Rwanda VMB study were at high risk of HIV/STI by design: 93.2% reported two or more partners in the month prior to the baseline visit and 76.6% reported no or inconsistent condom use. Much lower proportions of women in the HARP study reported these current sexual risk behaviors but they were all HIV-positive by design. Women in the HELIUS study were not selected based on sexual risk or HIV-status. The proportions of women reporting sexual risk behaviors could be considered average for a young, urban, Dutch population, but with differences by ethnic group: proportions were highest in women of Dutch origin, followed by Dutch women of sub-Saharan African (African Surinamese or Ghanaian) origin, and Dutch women of Turkish, Moroccan, or South Asian Surinamese origin. Current hormonal contraceptive use varied substantially between studies and ethnic groups, as did current smoking habits. Almost half of the women (39.4–45.1%; not assessed in the HARP study) reported current urogenital symptoms, but none of them had sought care for them. Laboratory-confirmed viral and bacterial STI prevalences were high in the Rwanda VMB study and low in the HELIUS study, whereas viral STI prevalences were high and bacterial STI prevalences low in the HARP study (consistent with high past but low current sexual risk). Antibiotic use in the 2 weeks prior to baseline was rare.

**Table 2:** Baseline characteristics by study, and by ethnic group within the HELIUS study.

	**VMB** **Rwanda** **(*N* = 162)**	**HARP** **South Africa** **(*N* = 455)**	**HELIUS Netherlands** **Sub-Saharan African origin^a^ (*N* = 183)**	**HELIUS Netherlands Turkish, Moroccan, South-Asian origin^b^ (*N* = 264)**	**HELIUS Netherlands** **Dutch origin** **(*N* = 99)**	**All studies** **(*N* = 1,163)**	***P-*value^c^**
Age (median, IQR)	30 (27–34)	34 (30–39)	26 (22–30)	27 (23–31)	26 (22-30)	30 (26–34)	<0.001
Currently pregnant (*n*%)^d^	6 (3.7)	0	0	0	0	6 (0.5)	<0.001
Contraceptive use if not pregnant (*n*%) - None or condom use only - Oral contraception^e^ - Progestin-only injectable - Progestin-only implant - Any intrauterine device^f^ - Contraceptive ring	*N = 156* 64 (41.0) 16 (10.3) 45 (28.9) 28 (18.0) 3 (1.9) 0	340 (74.7) 23 (5.1) 86 (18.9) 6 (1.3) 0 0	97 (53.6) 61 (33.7) 1 (0.6) 1 (0.6) 20 (11.1) 1 (0.6)	148 (56.3) 88 (33.5) 0 5 (1.9) 20 (7.6) 2 (0.8)	27 (27.7) 46 (46.5) 1 (1.0) 0 20 (20.2) 5 (5.1)	*N = 1,154* 676 (58.6) 234 (20.3) 133 (11.5) 40 (3.5) 63 (5.5) 8 (0.7)	<0.001
Currently using hormonal contraception or is pregnant (*n*%)^g^	95 (58.6)	115 (25.3)	*N = 161* 64 (39.8)	*N = 243* 95 (39.1)	*N = 79* 52 (65.8)	*N = 1,100* 421 (38.3)	<0.001
Current smoker (*n*%)	NA^h^	25 (5.5)	27 (14.8)	68 (25.9)	30 (30.3)	*N = 999* 150 (15.0)	<0.001
Used any antibiotic in past 14 days (*n*%)	0	NA	8 (4.4)	7 (2.7)	4 (4.0)	*N = 706* 19 (2.7)	0.024
Reported any type of vaginal cleansing (n%)^i^	*N = 64* 11 (17.2)	179 (39.3)	47 (25.8)	72 (27.7)	18 (18.2)	*N = 1,064* 327 (30.7)	<0.001
Number of sex partners in period prior to sampling^j^							
- None - One - Two or more	0 11 (6.8) 151 (93.2)	77 (17.0)352 (77.5)25 (5.5)	54 (29.7) 100 (55.0) 28 (15.4)	103 (39.0)142 (53.8)19 (7.2)	23 (23.2) 51 (51.5) 25 (25.3)	257 (22.1)656 (56.4)248 (21.3)	<0.001
Frequency of condom use (*n*%)^k^							
- Never - Inconsistent - Consistent - NA (no sexual partner)	9 (5.6) 115 (71.0) 38 (23.5) 0	24 (5.3) 129 (28.4) 221 (48.6) 81 (17.8)	60 (32.0) 42 (23.1) 26 (14.3) 54 (29.7)	99 (37.6) 39 (14.8) 22 (8.4) 103 (39.2)	43 (43.4) 26 (26.3) 7 (7.1) 23 (23.2)	235 (20.2) 351 (30.2) 314 (27.1) 262 (22.5)	<0.001
Reported any urogenital symptoms (*n*%)	73 (45.1)	NA	73 (39.9)	117 (44.3)	39 (39.4)	*N = 708* 302 (42.7)	0.644
Reported unusual vaginal discharge (*n*%)	21 (13.0)	NA	25 (13.7)	57 (21.6)	16 (16.2)	*N = 708* 119 (16.8)	0.069
Positive HIV test (*n*%)	16 (9.9)	455 (100)	0	0	0	471 (40.5)	<0.001
Yeasts by microscopy (*n*%)	*N = 140* 14 (10.0)	*N = 445* 34 (7.6)	NA	NA	NA	*N = 585* 48 (8.2)	0.380
*Trichomonas vaginalis* by culture/NAAT (*n*%)	*N = 138* 17 (12.3)	71 (15.6)	6 (3.3)	1 (0.4)	0	*N = 1,139* 95 (8.3)	<0.001
*Chlamydia trachomatis* by NAAT (*n*%)	*N = 139* 30 (21.6)	24 (5.3)	10 (5.5)	6 (2.3)	2 (2.0)	*N = 1,140* 72 (6.3)	<0.001
*Neisseria gonorrhoeae* by NAAT (*n*%)	*N = 139* 18 (13.0)	10 (2.2)	0	0	0	*N = 1,140* 28 (2.5)	<0.001
*Mycoplasma genitalium* by NAAT (*n*%)	NA	38 (8.4)	NA	NA	NA	NA	NA
Active syphilis by serology (*n*%)	13 (8.0)	*N = 452* 3 (0.7)	NA	NA	NA	*N = 614* 16 (2.6)	<0.001
Herpes simplex virus type 2 by serology (*n*%)	109 (67.3)	*N = 453*432 (95.4)	NA	NA	NA	*N = 615* 541 (88.0)	<0.001
Any high-risk HPV by PCR (*n*%)	NA	363 (79.8)	56 (30.6)	66 (25.0)	41 (41.4)	*N = 1,001* 526 (52.6)	<0.001

*HPV, human papilloma virus; IQR, inter-quartile range; NA, not applicable; NAAT, nucleic acid amplification test; PCR polymerase chain reaction*.

*The unit of analysis is one sample (collected at baseline) per woman*.

a *Included Dutch women of African Surinamese and Ghanaian origin*.

b *Included Dutch women of South-Asian Surinamese, Moroccan, and Turkish origin*.

c *Using the Fisher's exact test for binary variables, the Chi-squared test for categorical variables, and Kruskal-Wallis test for continuous variables*.

d *Pregnant women were not eligible for enrollment in any of the studies, but six women screened for the Rwanda VMB study were pregnant when the baseline vaginal swabs were taken, prior to enrollment*.

e *Includes combined and progestin-only oral contraception*.

f *In the VMB and HARP studies, only copper intrauterine devices were used. In the HELIUS study, women may have used either a copper or hormone-containing intrauterine device. One HELIUS participant used both an intrauterine device and a pill and she is included here*.

g *Excluding HELIUS participants who used intrauterine devices (including the participant who used an intrauterine device and a pill)*.

h *This question was not asked in the Rwanda VMB study but we know from previous studies in the same population that women rarely smoke*.

i *In the Rwanda VMB study, only participants who were subsequently randomized to the interventions were asked this question*.

j *The recall period was 1 month in the Rwanda VMB study, 3 months in the HARP study, and 6 months in the HELIUS study. In the Rwanda VMB study, the frequencies were as follows for 12 months recall: no partners 0%, one partner 2.5%, and two or more partners 97.5%*.

k *The recall period was 2 weeks in the Rwanda VMB study, 3 months in the HARP study, and 6 months in the HELIUS study*.

### Overall Vaginal Microbiota Characteristics

**Figure 1:**
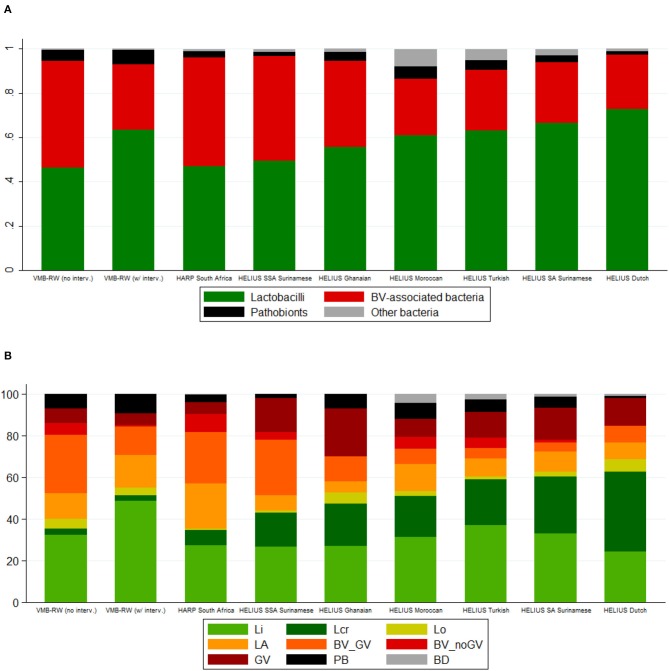
Bar charts by study, intervention exposure (VMB Rwanda) and ethnic group (HELIUS). **(A)** Mean relative abundance of bacterial groups. **(B)** Cumulative percentage of women with a specific VMB type. *BV*, bacterial vaginosis; *BD, Bifidobacterium*-dominated; *BV_GV*, polybacterial *Gardnerella vaginalis*-containing; *BV_noGV*, polybacterial but low *G. vaginalis*; *CI*, confidence interval; *GV, G. vaginalis*-dominated; *interv*, (study product) interventions; *LA*, lactobacilli and anaerobes; *Lcr, L. crispatus*-dominated; *Li, L. iners*-dominated; *Lo*, other lactobacilli-dominated; *NA*, not applicable; *PB*, pathobionts-containing; *SA*, South-Asian; *SSA*, sub-Saharan African; *VMB*, vaginal microbiota; *VMB-RW*, Rwanda VMB study. Rwanda VMB study samples collected at the screening and Month 6 visits in all randomization groups, and at the Month 1 and Month 2 visits in the no-intervention group, were considered not influenced by the interventions.

A heatmap of key taxa for all 2,044 samples from all three studies combined is shown in Supplementary Material 2, Table S1. In Table 3, Figure 1, VMB study samples were stratified by exposure to interventions, and HELIUS study samples by ethnic group. Mean Simpson diversity indexes and mean relative abundances of bacterial groups, and key taxa within these groups, differed significantly between the three studies and these pre-specified strata within studies (Table 3, Figure 1A). By far the most common bacterial groups in all studies and strata were the lactobacilli and BV-anaerobes, with mean relative abundances ranging from 0.46 to 0.73 and 0.25 to 0.49, respectively. The differences between studies and strata were as expected, with lower lactobacilli and higher BV-anaerobes proportions in women with higher sexual risk profiles and/or STI exposures and in women of sub-Saharan African ethnicities. In contrast, Rwanda VMB study participants who had recently been exposed to antibiotic/probiotic interventions had higher lactobacilli and lower BV-anaerobes proportions. The differences in *L. crispatus* mean relative abundance were especially striking, ranging from only 0.03 in the Rwanda VMB study samples that were not influenced by interventions to 0.38 in the HELIUS samples from women of Dutch origin. Mean relative abundances for pathobionts and the “other bacteria” group were low in all studies and strata, ranging from 0.01 to 0.07 and 0 to 0.05, respectively. The mean pathobionts relative abundance did not show a clear pattern between studies and strata but was lowest in the HELIUS women of Dutch origin. Estimated concentrations were only available for the Rwanda VMB study, and mean estimated concentrations in log_10_ cells/μl were 5.12 for lactobacilli (mostly consisting of *L. iners*), 5.17 for BV-anaerobes, 2.18 for pathobionts, and 1.92 for other bacteria in samples not influenced by interventions (Table 3). The mean pathobionts estimated concentration was therefore 871 times lower than the mean lactobacilli estimated concentration, and 977 times lower than the mean BV-anaerobes estimated concentration.

**Table 3:** Overview of VMB composition characteristics by study, stratified by intervention exposure (VMB Rwanda) and ethnic group (HELIUS).

	**VMB Rwanda** ** (not influenced by interventions)^a^**	**VMB Rwanda (influenced by interventions)^a^**	**HARP** ** South Africa**	**HELIUS** ** Sub-Saharan African origin^b^**	**HELIUS** ** Turkish, Moroccan, South-Asian origin^c^**	**HELIUS** ** Dutch origin**	**All groups**	***P*-value^d^**
Samples with relative abundance data available (*N*) Samples with estimated concentration data available (*N*)	366 158	263 221	869 0	183 0	264 0	99 0	2,044 379	NA
Nugent score category (n %)^e^: - 0–3 - 4–6 - 7–10	*N = 231* 79 (34.2) 27 (11.7) 125 (54.1)	*N = 227* 111 (48.9) 40 (17.6) 76 (33.5)	*N = 445* 151 (33.9) 102 (22.9) 192 (43.2)	NA	NA	NA	*N = 903* 341 (37.8) 169 (18.7) 393 (43.5)	<0.001
Simpson diversity index (1-D; mean, 95% CI)^f^	0.53 (0.50–0.56)	0.40 (0.36–0.44)	0.54 (0.52–0.56)	0.39 (0.35–0.43)	0.34 (0.31–0.38)	0.32 (0.27–0.37)	0.47 (0.46–0.48)	<0.001
VMB types (*n* %)^g^: - *L. iners*-dominated (Li) - *L. crispatus*-dominated (Lcr) - Dominated by other lactobacilli (Lo) - Lactobacilli plus anaerobes (LA) - Polybacterial *G. vaginalis*-containing (BV_GV) - Polybacterial with little *G. vaginalis* (BV_noGV) - *G. vaginalis*-dominated (GV) - Pathobionts-containing (PB)	119 (32.5) 10 (2.7) 18 (4.9) 45 (12.3) 102 (27.9) 21 (5.7) 26 (7.1) 25 (6.8)	128 (48.7) 7 (2.7) 10 (3.8) 41 (15.6) 36 (13.7) 2 (0.8) 15 (5.7) 24 (9.1)	237 (27.3) 65 (7.5) 5 (0.6) 188 (21.6) 215 (24.7) 75 (8.6) 49 (5.6) 33 (3.8)	49 (26.8) 33 (18.0) 5 (2.7) 12 (6.6) 38 (20.8) 4 (2.2) 35 (19.1) 7 (3.8)	89 (34.6) 61 (23.7) 5 (2.0) 28 (10.9) 15 (5.8) 10 (3.9) 32 (12.5) 17 (6.6)	24 (24.5) 38 (38.8) 6 (6.1) 8 (8.2) 8 (8.2) 0 13 (13.3) 1 (1.0)	646 (31.6) 214 (10.5) 49 (2.4) 322 (15.8) 414 (20.3) 112 (5.5) 170 (8.3) 107 (5.2)	<0.001
Relative abundance of VMB bacterial groups (mean, 95% CI):								
- Total lactobacilli - *L. iners* - *L. crispatus* - Other lactobacilli - Total BV-anaerobes - *G. vaginalis* - *A. vaginae* - *Prevotella* species - Other BV-anaerobes - Total pathobionts - *Streptococcus* species -*Staphylococcus* species - *Escherichia/Shigella* species - Other pathobionts^h^^,^^i^ - Total other bacteria	0.46 (0.42–0.51) 0.36 (0.32–0.40) 0.03 (0.02–0.05) 0.07 (0.05–0.09) 0.48 (0.44–0.52) 0.18 (0.16-0.20) 0.03 (0.02-0.04) 0.07 (0.06–0.08) 0.20 (0.18–0.23) 0.05 (0.03–0.07) 0.04 (0.02–0.05) 0 (0–0.01) 0.01 (0–0.02) 0 (0–0) 0 (0–0)	0.63 (0.59–0.68) 0.53 (0.48–0.58) 0.04 (0.02–0.06) 0.07 (0.04–0.09) 0.30 (0.25–0.34) 0.15 (0.12–0.17) 0.01 (0.01–0.02) 0.03 (0.02–0.04) 0.10 (0.08–0.12) 0.07 (0.04–0.09) 0.05 (0.03–0.07) 0 (0–0) 0.01 (0–0.02) 0.01 (0–0.01) 0 (0–0.01)	0.47 (0.44–0.50) 0.37 (0.34–0.39) 0.08 (0.07–0.10) 0.02 (0.01–0.02) 0.49 (0.46–0.52) 0.17 (0.15–0.18) 0.03 (0.02–0.03) 0.06 (0.06–0.07) 0.23 (0.22–0.25) 0.03 (0.02–0.04) 0.02 (0.01–0.03) 0 (0–0) 0 (0–0) 0 (0–0) 0.01 (0.01–0.01)	0.52 (0.45–0.58) 0.30 (0.25–0.36) 0.18 (0.13–0.23) 0.04 (0.02–0.06) 0.44 (0.37–0.50) 0.23 (0.19–0.27) 0.09 (0.07–0.11) 0.02 (0.01–0.03) 0.10 (0.07–0.12) 0.03 (0.01–0.05) 0.02 (0.01–0.04) 0 (0–0) 0 (0–0.01) 0 (0–0) 0.01 (0.01–0.02)	0.64 (0.59–0.69) 0.34 (0.29–0.39) 0.24 (0.19–0.28) 0.06 (0.04–0.08) 0.27 (0.22–0.31) 0.16 (0.12–0.19) 0.04 (0.03–0.05) 0.02 (0.01–0.03) 0.05 (0.04–0.06) 0.04 (0.02–0.06) 0.03 (0.02–0.04) 0 (0–0) 0 (0–0.01) 0.01 (0–0.01) 0.05 (0.03–0.07)	0.73 (0.65–0.80) 0.28 (0.20–0.35) 0.38 (0.29–0.46) 0.08 (0.04–0.11) 0.25 (0.17–0.32) 0.16 (0.10–0.21) 0.06 (0.03–0.08) 0.01 (0–0.01) 0.03 (0.02–0.04) 0.01 (0–0.03) 0 (0–0.01) 0.01 (0–0.03) 0 (0–0) 0 (0–0) 0.01 (0.00–0.03)	0.53 (0.51–0.55) 0.37 (0.36–0.39) 0.11 (0.10–0.12) 0.04 (0.04–0.05) 0.42 (0.40–0.44) 0.17 (0.16–0.18) 0.04 (0.03–0.04) 0.05 (0.04–0.05) 0.16 (0.15–0.17) 0.04 (0.03–0.04) 0.03 (0.02–0.03) 0 (0–0) 0 (0–0.01) 0 (0–0) 0.01 (0.01–0.02)	<0.001 <0.001 <0.001 <0.001 <0.001 <0.001 <0.001 <0.001 <0.001 <0.001 <0.001 <0.001 <0.001 <0.001 <0.001
Relative abundance total pathobionts categorical (n %):								
<1% of reads 1%–<10% 10%–<20% 20%–<50% 50% or more	467 (74.2) 87 (13.8) 26 (4.1) 24 (3.9) 25 (4.0)	190 (72.2) 36 (13.7) 13 (4.9) 10 (3.8) 14 (5.3)	726 (83.5) 88 (10.1) 22 (2.5) 18 (2.1) 15 (1.7)	162 (88.5) 14 (7.7) 0 2 (1.1) 5 (2.7)	203 (76.9) 42 (15.9) 2 (0.8) 10 (3.8) 7 (2.7)	87 (87.9) 10 (10.1) 1 (1.0) 0 1 (1.0)	1,645 (80.5) 241 (11.8) 51 (2.5) 54 (2.6) 53 (2.6)	<0.001
Estimated concentration of VMB bacterial groups in log_10_ cells/μl (mean, 95% CI):							
- Total lactobacilli -*L. iners* - *L. crispatus*-Other lactobacilli - Total BV-anaerobes -*G. vaginalis* - *A. vaginae* - *Prevotella* species - Other BV-anaerobes - Total pathobionts - *Streptococcus* species -*Staphylococcus* species -*Enterococcus* species -*Escherichia/Shigella* species -*Campylobacter* species -*Haemophilus* species - Other pathobionts^i^^,^^j^ - Total other bacteria	5.12 (4.97–5.27) 4.78 (4.60–4.97) 0.64 (0.48–0.80) 2.29 (2.08–2.50) 5.17 (4.99–5.35) 4.48 (4.25–4.71) 2.80 (2.53–3.07) 3.00 (2.75–3.25) 4.30 (4.09–4.51) 2.18 (1.96–2.40) 1.59 (1.38–1.81) 0.46 (0.35–0.58) 0.17 (0.09–0.25) 0.50 (0.37–0.63) 0.12 (0.05–0.19) 0.07 (0.02–0.12) 0.36 (0.18–0.53) 1.92 (1.72–2.11)	4.84 (4.58–5.10) 4.56 (4.25–4.87) 0.44 (0.23–0.65) 1.73 (1.40–2.06) 5.74 (5.48–6.01) 5.08 (4.75–5.40) 3.97 (3.56–4.38) 4,01 (3.65–4.38) 5.08 (4.79–5.37) 2.04 (1.69–2.38) 1.47 (1.14–1.80) 0.41 (0.24–0.58) 0.06 (0–0.13) 0.35 (0.17–0.54) 0.17 (0.04–0.29) 0.08 (0–0.16) 0.40 (0.25–0.55) 1.92 (1.61–2.31)	NA	NA	NA	NA	5.32 (5.15–5.49) 4.95 (4.72–5.17) 0.78 (0.56–1.01) 2.69 (2.43–2.96) 4.76 (4.53–4.99) 4.05 (3.75–4.35) 1.96 (1.64–2.29) 2.28 (1.97–2.59) 3.75 (3.48–4.02) 2.28 (1.99–2.57) 1.68 (1.39–1.97) 0.50 (0.34–0.66) 0.24 (0.12–0.37) 0.61 (0.42–0.79) 0.09 (0.01–0.17) 0.07 (0–0.13) 0.38 (0.27–0.49) 1.92 (1.66–2.17)	0.005 0.051 0.049 <0.001 <0.001 <0.001 <0.001 <0.001 <0.001 0.318 0.326 0.400 0.055 0.030 0.239 0.860 0.579 1.00

*A, Atopobium; BV, bacterial vaginosis; CI, confidence interval; G, Gardnerella; L, Lactobacillus; NA, not assessed/applicable; VMB, vaginal microbiota*.

*The unit of analysis is one sample. Cells contain at most five missing values unless otherwise indicated*.

a *Samples collected at the screening and Month 6 visits in all randomization groups, and at the Month 1 and Month 2 visits in the no–intervention group, were considered not influenced by the interventions*.

b *Included Dutch women of African Surinamese and Ghanaian origin*.

c *Included Dutch women of South–Asian Surinamese, Moroccan, and Turkish origin*.

d *Using the Chi-squared test for categorical variables and the Kruskal-Wallis test for continuous variables*.

e *Nugent scoring of Gram stains was performed during the all scheduled study visits in the VMB study, the first study visit in the HARP study, and not at all in the HELIUS study*.

f *Based on the rarefied sequencing data set of each of the studies*.

g *The HARP and HELIUS studies also identified samples that had significant abundance of Bifidobacteria (n = 2 in HARP and n = 8 in HELIUS)*.

h *These pathobiont genera were uncommon (mean relative abundance lower than 1% for each of the genera)*.

i *Also includes reads assigned to the pathogens Chlamydia, Neisseria, and Treponema genus*.

j *Individual pathobionts in this rest group were detected at a mean estimated concentration of at most 0.02 log_10_ cells/μl in the Rwanda VMB study*.

The VMB types for all samples combined (*N* = 2,044) were distributed as follows: Li 31.6%, Lcr 10.5%, Lo 2.4%, LA 15.8%, BV_GV 20.3%, BV_noGV 5.5%, GV 8.3%, PB 5.2%, and BD 0.5%. The latter VMB type included only 10 samples and was therefore not included in subsequent comparisons. Consistent with the bacterial group findings, the VMB types characterized by lactobacilli-domination (Li, Lcr, and Lo; 44.5%) or by anaerobic dysbiosis (LA, BV_GV, BV_noGV, GV; 49.9%) were much more common than the VMB type characterized by ≥20% pathobionts (5.2%). VMB type distributions differed significantly between the studies and strata, following the same patterns as described above for the bacterial group findings (Table 3, Figure 1B).

### Identification of Common Vaginal Pathobionts

We identified 40 different pathobiont taxa in all 2,044 samples combined (Supplementary Material 1; reported at species level if only one species was identified, genus level if multiple species and/or the genus was identified, and family or class level if only that level was identified). However, 20 of these were never a non-minority taxon (defined as present at a relative abundance of at least 1% in at least one sample) in any of the studies. Only six taxa were a non-minority taxon in all three studies: *Streptococcus, Staphylococcus, Enterococcus, Escherichia/Shigella, Haemophilus*, and *Campylobacter*. *Chlamydia* was consistently detected in all three studies but only as a non-minority taxon in HELIUS, and *Neisseria* and *Treponema* were detected in the two African studies only. The remaining 11 taxons varied in their detection (yes vs. no) and relative abundance (minority vs. non-minority) status between the three studies. More than half (54%) of all pathobiont sequencing reads were assigned to *Streptococcus* genus/species and 24% of the *Streptococcus* reads were assigned to *S. agalactiae* or *S. agalactiae/pyogenes*. Seventeen percent of all women (196/1,153) at baseline, and 19.5% (399/2,044) of all samples, had at least 1% pathobionts in their VMB; these proportions were 12.7% (147/1,153), and 14.9% (304/2,044) for at least 1% *Streptoccoccus*. Among samples with ≥20% pathobionts (*N* = 107; Supplementary Material 2, Table S1), 33 contained *Streptococcus* genus/species as the only pathobionts (relative abundances of 0.53–0.98) and an additional 52 contained multiple pathobionts including substantial relative abundances of *Streptococcus* genus/species (0.13–0.73). The other 22 samples contained other pathobionts (most commonly staphylococci, *Escherichia/Shigella* species, *Haemophilus* species, and/or enterococci), with <5% *Streptococcus*.

Of note, the total estimated bacterial concentration differed significantly per VMB type (Table 4; data available for the Rwanda VMB study only). The mean total bacterial estimated concentration of women with the PB VMB type (5.85 log_10_ cells/μl) was comparable to those of women with *Lactobacillus*-dominated VMB types (5.13–5.83 log_10_ cells/μl) but lower than those of women with VMB types associated with anaerobic dysbiosis (6.11–6.87 log_10_ cells/μl). When samples were stratified by the proportion of pathobionts in the VMB (<1%, 1- <10%, 10%- <20%, 20- <50%, and ≥50%), the mean estimated concentration of pathobionts increased as expected, but reached a plateau at proportions of 10% or more. The mean estimated concentration of total bacteria remained stable but declined somewhat when the pathobionts proportion reached above 20%. Results were similar when only samples not influenced by interventions were included in these analyses (Table 4).

**Table 4:** Estimated bacterial concentration per VMB type and by proportion of pathobionts (Rwanda VMB study only, multiple samples per woman).

**VMB type**		**Estimated total bacterial concentration in log**_****10****_ **cells/μl**		
	**N**	**All samples** **Mean (95% CI)^b^**	**N**	**Not influenced by interventions^a^** **Mean (95% CI)^**c**^**
*Lactobacillus iners*-dominated (Li)	144	5.81 (5.69–5.92)	34	5.99 (5.75–6.22)
*L. crispatus*-dominated (Lcr)	9	5.36 (4.99–5.72)	3	5.43 (4.51–6.35)
Other lactobacilli-dominated (Lo)	10	5.13 (4.63–5.64)	3	5.59 (4.13–7.05)
Lactobacilli and BV-anaerobes (LA)	57	6.31 (6.11–6.51)	21	6.34 (5.96–6.73)
Polybacterial *Gardnerella vaginalis*-containing (BV_GV)	90	6.87 (6.72–7.02)	57	6.91 (6.72–7.11)
Polybacterial but low *G. vaginalis* (BV_noGV)	16	6.29 (5.75–6.82)	14	6.15 (5.57–6.73)
*G. vaginalis*-dominated (GV)	27	6.11 (5.77–6.46)	16	6.14 (5.64–6.64)
Pathobionts-containing (PB)	26	5.85 (5.43–6.27)	10	5.76 (4.76–6.76)
**Proportion pathobionts**		**Estimated total bacterial concentration in log**_**10**_ **cells/μl**		
<1%	289	6.17 (6.07–6.27)	124	6.47 (6.31–6.62)
1%–<10%	50	6.14 (5.89–6.38)	19	6.05 (5.69–6.42)
10%–<20%	14	6.33 (5.89–6.78)	5	6.29 (5.02–7.56)
20%–<50%	15	6.04 (5.49–6.59)	8	6.18 (5.22–7.15)
≥50%	11	5.59 (4.84–6.34)	2	4.06 (−5.20–13.33)
**Proportion pathobionts**		**Estimated pathobionts concentration in log**_**10**_ **cells/μl**		
<1%	289	1.36 (1.16–11.56)	124	1.32 (0.99–1.65)
1%–<10%	50	4.38 (4.12–4.63)	19	4.21 (3.84–4.58)
10%–<20%	14	5.25 (4.80–5.69)	5	5.21 (4.00–6.41)
20%–<50%	15	5.40 (4.85–5.94)	8	5.51 (4.51–6.50)
≥50%	11	5.41 (4.71–6.12)	2	4.00 (−6.02–14.02)

*BV, bacterial vaginosis; CI, confidence interval; VMB, vaginal microbiota*.

a *Estimated concentrations were only available for the Rwanda VMB study. Samples collected at the screening and Month 6 visits in all randomization groups, and at the Month 1 and Month 2 visits in the no-intervention group, were considered not influenced by the interventions*.

b *The Kruskall Wallis p-values comparing the relevant categories are <0.001 for VMB types and estimated total bacterial concentration, 0.606 for pathobionts proportion and estimated total bacterial concentration, and <0.001 for pathobionts proportion and estimated pathobionts concentration*.

*The Kruskall Wallis p-value comparing the relevant categories are <0.001 for VMB types, 0.061 for pathobionts proportion and estimated total bacterial concentration, and <0.001 for pathobionts proportion and estimated pathobionts concentration*.

### Correlations Between Vaginal Pathobionts and Other VMB Characteristics

We next investigated correlations between pathobionts levels (relative abundances or estimated concentrations), lactobacilli levels, and BV-anaerobes levels for samples not influenced by interventions (Table 5). With increasing pathobionts proportion (from <1% to 1- <10% to 10%- <20%, etc.), the mean relative abundance of lactobacilli declined significantly (ρ = −0.1851; 95% confidence interval (CI) −0.2286 to −0.1416). The same applied to estimated concentrations, but this trend was not significant (ρ = −0.0132; 95% CI −0.1891 to 0.1627). We could not detect a pathobionts proportion threshold: the weak negative effect on lactobacilli was detectable even in the lowest pathobionts proportion categories. By contrast, the mean relative abundance of BV-anaerobes remained stable initially, and only declined when pathobionts made up 30% or more of the VMB. The mean BV-anaerobes estimated concentration significantly increased with increasing estimated pathobionts concentration and did not reach a plateau. In all pathobionts concentration categories, BV-anaerobes outnumbered pathobionts.

**Table 5:** VMB correlates by pathobionts relative abundance category, all samples from all studies not influenced by interventions (*N* = 1,781)^a^.

**Cells: mean relative abundance (95% CI)**	**Total pathobionts relative abundance category**							**ρ (95% CI)**	**r (95% CI)**
	**<1%**	**1%–<10%**	**10%–<20%**	**20%–<30%**	**30%–<40%**	**40%–<50%**	**≥50%**	**All ^b^**	**If ≥1%^c^**
	**(*n* = 1,445)**	**(*n* = 205)**	**(*n* = 38)**	**(*n* = 15)**	**(*n* = 15)**	**(*n* = 14)**	**(*n* = 39)**	**(*N* = 1,781)**	**(*n* = 326)**
Total pathobionts	0 (0–0)	0.03 (0.03–0.03)	0.15 (0.14–0.16)	0.25 (0.23–0.27)	0.35 (0.33–0.37)	0.46 (0.44–0.47)	0.81 (0.76–0.85)	NA	NA
Total lactobacilli	0.55 (0.53–0.57)	0.45 (0.39–0.50)	0.25 (0.14–0.36)	0.21 (0.05–0.36)	0.24 (0.10–0.39)	0.15 (0.05–0.25)	0.07 (0.03–0.10)	−0.1851 (−0.2286, −0.1416)	−0.3756 (−0.4340,−0.3172)
Total BV-anaerobes	0.44 (0.42–0.46)	0.46 (0.41–0.51)	0.52 (0.41–0.64)	0.53 (0.38–0.68)	0.33 (0.20–0.46)	0.35 (0.24–0.46)	0.10 (0.06–0.13)	−0.0012 (−0.0453, 0.0429)	−0.3223 (-0.3946,−0.2501)
Total other bacteria	0.01 (0.00–0.01)	0.06 (0.04–0.09)	0.08 (0.01–0.15)	0.01 (0.00–0.02)	0.08 (0.00–0.15)	0.04 (0.00–0.07)	0.02 (0.01–0.04)	0.3894 (0.3464, 0.4323)	−0.0866 (−0.1356, −0.0376)
*Lactobacillus iners*	0.37 (0.35–0.39)	0.32 (0.27–0.36)	0.21 (0.11–0.32)	0.18 (0.04–0.32)	0.17 (0.04–0.31)	0.08 (-0.01–0.16)	0.04 (0.01–0.07)	−0.1564 (−0.2020, −0.1106)	−0.3069 (-0.3584,−0.2553)
*L. crispatus*	0.13 (0.12–0.15)	0.10 (0.07–0.13)	0.03 (−0.01–0.08)	0.01 (−0.01–0.03)	0.02 (0.00–0.04)	0.05 (−0.01–0.12)	0.02 (0.00–0.04)	0.0470 (0.0022, 0.0919)	−0.1484 (−0.1953, −0.1061)
Other lactobacilli	0.04 (0.04–0.05)	0.03 (0.02–0.04)	0.01 (0.00–0.01)	0.02 (0.00–0.03)	0.05 (−0.03–0.14)	0.02 (−0.01–0.06)	0.01 (−0.01–0.03)	0.0397 (−0.0272, 0.5989)	−0.0700 (−0.1290, −0.0010)
*Gardnerella vaginalis*	0.18 (0.17–0.19)	0.17 (0.14–0.20)	0.20 (0.12–0.28)	0.29 (0.12–0.45)	0.17 (0.05–0.29)	0.15 (0.06–0.25)	0.04 (0.01–0.07)	0.0038 (−0.0408, 0.0485)	−0.1759 (−0.2390, −0.1129)
*Atopobium vaginae*	0.04 (0.04–0.05)	0.02 (0.01–0.03)	0.02 (0.00–0.03)	0.04 (−0.02–0.11)	0.00 (0.00–0.01)	0.01 (0.00–0.02)	0 (0–0.01)	−0.1102 (−0.1560, −0.0643)	−0.1032 (−0.1567, −0.0496)
*Prevotella* species	0.05 (0.04–0.05)	0.07 (0.05–0.08)	0.09 (0.04–0.14)	0.03 (0.00–0.05)	0.05 (0.00–0.09)	0.04 (0.01–0.06)	0.01 (0.00–0.02)	0.0551 (0.0077, 0.1025)	−0.1961 (−0.2490, −0.1433)
Other BV-anaerobes	0.17 (0.16–0.19)	0.20 (0.17–0.24)	0.21 (0.15–0.28)	0.18 (0.06–0.30)	0.11 (0.04–0.19)	0.15 (0.05–0.25)	0.04 (0.02–0.06)	0.0504 (0.0040, 0.0968)	−0.2382 (−0.2994, −0.1771)
**Cells: mean estimated concentration in log**_**10**_ **cells/μL (95% CI)**^d^	** <10**^**3**^	**10**^**3**^**- <10**^**4**^	**10**^**4**^**- <10**^**5**^	**10**^**5**^**- <10**^**6**^	**≥10**^**6**^			**ρ (95% CI)** **All**^b^	**r (95% CI)** **If** **≥1%**^c^
	**(*****n*** **=** **89)**	**(*****n*** **=** **33)**	**(*****n*** **=** **21)**	**(*****n*** **=** **10)**	**(*****n*** **=** **5)**			**(*****n*** **=** **158)**	**(*****n*** **=** **34)**
Total pathobionts	0.25 (0.09–0.40)	3.59 (3.48–3.70)	4.51 (4.37–4.65)	5.40 (5.23–5.56)	6.50 (5.92–7.09)			NA	NA
Total bacteria	6.30 (6.10–6.49)	6.12 (5.83–6.42)	6.45 (6.00–6.91)	7.04 (6.43–7.65)	7.46 (7.18–7.74)			0.1219 (−0.0374, 0.2811)	0.8117 (0.7200, 0.9034)
Total lactobacilli	4.98 (4.70–5.26)	4.68 (4.12–5.24)	4.66 (3.71–5.62)	5.22 (3.68–6.76)	3.47 (−0.62–7.67)			−0.0132 (−0.1891, 0.1627)	0.0366 (−0.3937, 0.4669)
Total BV-anaerobes	5.60 (5.21–5.98)	5.55 (5.02–6.08)	5.88 (5.20–6.57)	6.68 (5.77–7.60)	7.22 (6.69–7.76)			0.1009 (−0.0596, 0.2615)	0.5778 (0.3391, 0.8166)
Total other bacteria	1.43 (1.05–1.81)	2.67 (2.05–3.28)	2.37 (1.41–3.33)	2.76 (0.99–4.52)	2.19 (−1.60–5.98)			0.2652 (0.1026, 0.4278)	0.0689 (−0.3669, 0.5048)
*L. iners*	4.65 (4.28–5.03)	4.31 (3.65–4.98)	4.54 (3.51–5.58)	5.22 (3.68–6.76)	3.29 (−0.60–7.19)			−0.0115 (−0.1817, 0.1588)	0.0891 (−0.3009, 0.4791)
*L. crispatus*	0.47 (0.19–0.74)	0.53 (0.02–1.05)	0.20 (−0.22–0.63)	0 (0–0)	1.28 (−2.28–4.85)			0.0325 (−0.1103, 0.1753)	0.2864 (−0.2120, 0.7849)
Other lactobacilli	1.56 (1.14–1.98)	1.98 (1.25–2.72)	2.24 (1.24–3.24)	0.97 (−0.49–2.43)	2.38 (−1.69–6.44)			0.0975 (−0.0672, 0.2622)	0.0767 (−0.3394, 0.4927)
*G. vaginalis*	4.98 (4.54–5.43)	5.03 (4.42–5.64)	5.13 (4.17–6.10)	5.82 (4.18–7.45)	5.41 (1.63–9.20)			0.0657 (−0.1036, 0.2350)	0.1127 (−0.3010, 0.5264)
*A. vaginae*	4.36 (3.86–4.86)	3.60 (2.72–4.47)	3.21 (1.84–4.58)	3.87 (1.45–6.30)	2.81 (−1.97–7.58)			−0.1391 (−0.3162, 0.0380)	−0.0221 (−0.3908, 0.3464)
*Prevotella* species	4.14 (3.67–4.62)	3.68 (2.92–4.44)	3.33 (2.05–4.61)	4.77 (2.69–6.84)	5.15 (3.88–6.41)			−0.0384 (−0.2086, 0.1317)	0.4183 (0.1589, 0.6778)
Other BV-anaerobes	4.87 (4.43–5.31)	4.87 (4.34–5.41)	5.34 (4.68–6.01)	6.25 (5.41–7.09)	6.70 (5.85–7.54)			0.1090 (−0.0496, 0.2675)	0.6760 (0.4868, 0.8653)

*BV, bacterial vaginosis; CI, confidence interval; NA, not applicable; VMB, vaginal microbiota*.

a *VMB study samples collected at the screening and Month 6 visits in all randomization groups, and at the Month 1 and Month 2 visits in the no-intervention group, were considered not influenced by the interventions*.

b *Spearman's rank correlation coefficient calculations included all samples not influenced by interventions*.

c *Pearson's correlation coefficient calculations only included samples not influenced by interventions and containing at least 1% pathobionts*.

d *Estimated concentrations are available for Rwanda VMB study samples only*.

**Figure 2:**
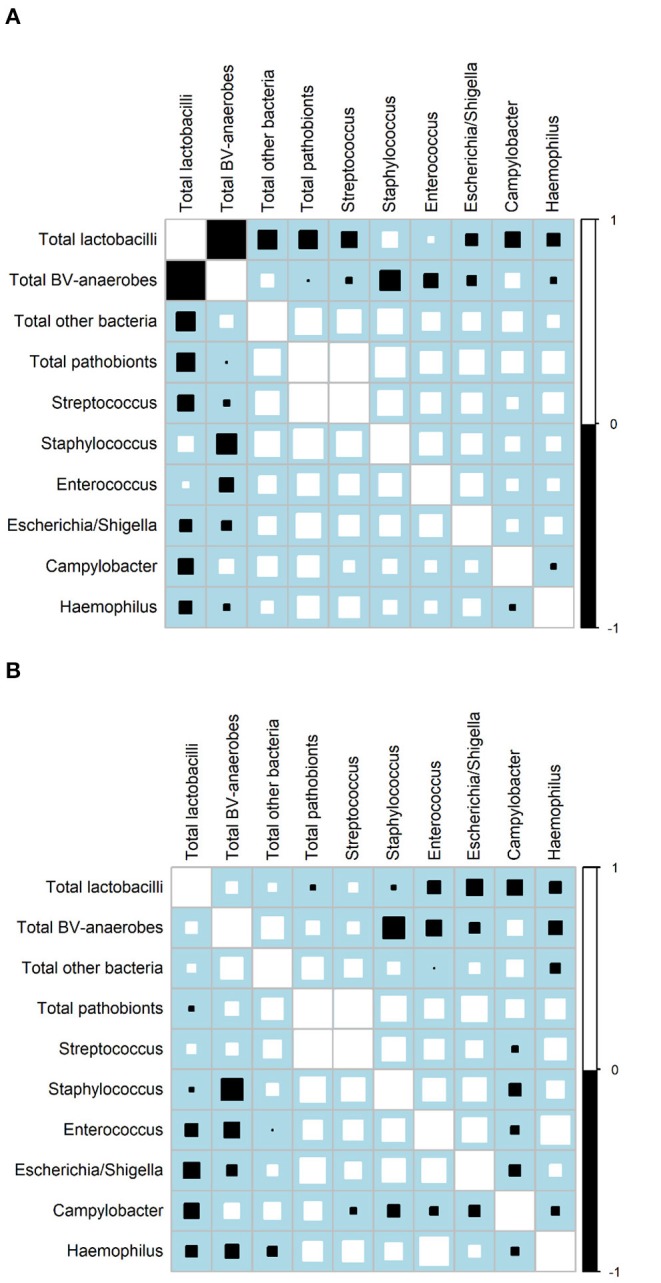
Correlation matrices. **(A)** Relative abundances, all three studies (*N* = 1,781 samples)^*a*^. **(B)** Estimated concentrations, VMB study only (*N* = 158 samples)^*a*^. *BV*, bacterial vaginosis.^*a*^Only includes VMB samples not influenced by the interventions, collected at Screening, Month 6, and Month 1 and 2 in the no-intervention group. Squares indicate the Spearman's rank correlation between −1 and +1. Positive correlations are shown in white and negative correlations in black. The size of each square is loosely proportionate to the magnitude of the correlation. For the actual correlation coefficients, (see Supplementary Material, Tables S2C,D).

Correlation matrixes for samples not influenced by interventions confirmed that relative abundances of BV-anaerobes and lactobacilli were strongly negatively correlated (ρ = −0.9234; Figure 2A), but showed that their estimated concentrations were not correlated (*r* = 0.0031; Figure 2B; for correlation coefficients with 95% confidence intervals, see Supplementary Material 2, Table S2). Pathobionts and lactobacilli relative abundances were also negatively correlated, albeit less strongly (ρ = −0.2076), and their estimated concentrations were not (*r* = 0.0436). Pathobionts and BV-anaerobes relative abundances were not correlated (ρ = 0.0160) and their estimated concentrations were weakly positively correlated (*r* = 0.1938). Pathobionts also correlated positively with the “other bacteria” rest group (ρ = 0.3831 for relative abundances and *r* = 0.3388 for estimated concentrations). At individual genus level, the estimated concentrations of the six pathobionts that were a non-minority genus in all three studies correlated positively with one another except for *Campylobacter* with the other five taxa, and *Haemophilus* and *Escherichia/Shigella*.

### Correlates of Vaginal Pathobionts Detection

Finally, we investigated the correlates of pathobionts detection (≥1% vs. <1%), relative abundance, and estimated concentration for all studies combined (Table 6) and for each study separately (Supplementary Material 2, Tables S3A–C). The mean relative abundance and mean estimated concentration of pathobionts decreased with increasing age (except in the HELIUS study), and with ethnicities other than Dutch. The data consistently showed strong associations with Nugent score categories in both studies that assessed these (the Rwanda VMB and HARP studies): the likelihood of detection (OR = 5.29; 95% CI 2.82–9.90), mean relative abundance, and mean estimated concentration of pathobionts were highest for Nugent score category 4–6 (intermediate), followed by 7–10 (BV-positive), and 0–3 (BV-negative). Positive associations between pathobionts detection or levels and hormonal contraceptive use, smoking, antibiotic use in the 14 days prior to sampling, HIV status, and the presence of STI pathogens were found in at least one but not in all studies. Associations with sexual risk-taking and unusual vaginal discharge reporting were inconsistent between studies, and we did not find associations with detection of vaginal yeasts by microscopy.

**Table 6:** Correlates of pathobionts detection, relative abundance, and estimated concentration.

	**Rwanda VMB study screening, HARP baseline, and HELIUS samples**				**All samples**			
**Independent variables^a^**	** <1%** **(% of *N* = 957)**	**≥1%** **(% of *N* = 196)**	**OR (95% CI)^b^**	***P*^c^**	**Mean relative abundance (95% CI); *N* = 2,044**	***P*^c^**	**Mean estimated concentration log_**10**_ cells/μl (95% CI); *N* = 379**	***P*^c^**
Potentially influenced by interventions:^d^								
- Yes - No	NA^d^	NA^d^	NA^d^	NA^d^	0.07 (0.04–0.09) 0.03 (0.03–0.04)	**0.032**	2.28 (1.99–2.57) 2.04 (1.69–2.38)	0.318
Age categories: - 18–24 - 25–29 - 30–34 - 35–44 - 45–50	19.8 25.7 31.5 20.5 2.6	21.4 27.6 29.6 17.9 3.6	Reference 0.99 (0.63–1.54) 0.87 (0.56–1.34) 0.80 (0.49–1.31) 1.26 (0.51–3.11)	0.719	0.08 (0.06–0.11) 0.04 (0.03–0.05) 0.03 (0.02–0.03) 0.03 (0.02–0.04) 0.02 (0.01–0.04)	** <0.001**	3.09 (2.56–3.62) 2.29 (1.80–2.78) 2.01 (1.66–2.35) 1.63 (1.19–2.06) NA	** <0.001**
Study or ethnicity: - VMB Rwanda - HARP South Africa - HELIUS sub - Saharan African - HELIUS Turkish, Moroccan, South - Asian - HELIUS Dutch	14.2 38.6 16.9 21.2 9.1	13.3 38.8 10.7 31.1 6.1	1.39 (0.66–2.89) 1.49 (0.78–2.87) 0.94 (0.44–2.00) 2.18 (1.12–4.25) Reference	**0.013**	0.06 (0.04–0.07) 0.03 (0.02–0.04) 0.03 (0.01–0.05) 0.04 (0.03–0.06) 0.01 (0.00–0.03)	** <0.001**	2.18 (1.96–2.40) NA NA NA NA	NA
Contraceptive use: - None or condom use only - Any oral contraception - Progestin-only injectable - Progestin-only implant - Any IUD (copper or hormonal) - Contraceptive ring - NA (currently pregnant)	*N = 954* 58.0 20.7 11.9 3.1 5.7 0.6 0.6	61.2 19.4 8.2 5.6 4.6 1.0 0	Reference 0.89 (0.60–1.33) 0.65 (0.37–1.14) 1.74 (0.84–3.58) 0.76 (0.37–1.59) 1.53 (0.30–7.66) ND	0.309	*N = 1,768* 0.03 (0.02–0.03) 0.03 (0.02–0.04) 0.05 (0.03–0.07) 0.04 (0.02–0.06) 0.01 (0.00–0.03) 0.02 (−0.02–0.05) 0 (0–0)	**0.026**	*N = 252* 1.53 (1.11–1.95) 2.89 (1.93–3.86) 2.37 (1.86–2.88) 2.58 (2.00–3.17) 1.55 (0.05–3.04) NA 1.27 (−4.21–6.75)	**0.014**
Any hormonal contraception or pregnant: - Yes - No	*N = 902* 38.4 61.6	*N = 188* 35.6 64.4	0.87 (0.63–1.21) Reference	0.415	*N = 1,768* 0.04 (0.03–0.05) 0.03 (0.02–0.03)	**0.026**	*N = 252* 2.50 (2.15–2.85) 1.53 (1.13–1.93)	** <0.001**
Current smoker: - Yes - No	*N = 819* 15.5 84.5	*N = 170* 13.5 86.5	0.85 (0.53–1.38) Reference	0.508	*N = 1,413* 0.01 (0.00–0.03) 0.03 (0.02–0.04)	0.960	NA	NA
Sample taken:^e^ - During or within 7 days after menses - Not during or within 7 days after menses	NA	NA	NA	NA	*N = 462* 0.05 (0.02–0.09) 0.08 (0.06–0.10)	0.236	*N = 310* 2.12 (1.63–2.62) 2.25 (1.97–2.53)	0.606
Any type of vaginal cleansing: - Yes - No	*N = 871* 30.7 69.4	*N = 190* 27.9 72.1	0.88 (0.62–1.24) Reference	0.462	*N = 1,320* 0.03 (0.01–0.04) 0.04 (0.03–0.05)	0.805	*N = 310* 2.47 (1.82–3.11) 2.18 (1.92–2.44)	0.417
Number of sex partners prior to sampling: - None - One - Two or more	21.4 57.1 21.4	25.6 52.3 22.1	Reference 0.77 (0.53–1.11) 0.86 (0.55–1.35)	0.379	*N = 1,775* 0.04 (0.02–0.06) 0.03 (0.01–0.03) 0.04 (0.02–0.05)	**0.008**	*N=249* 1.81 (−21.19–24.82) 1.82 (1.21–2.43) 2.17 (1.87–2.48)	0.571
Frequency of condom use: - Never - Inconsistent - Consistent - NA (no sexual partner)	20.5 30.8 26.8 21.9	18.4 28.6 27.6 25.5	Reference 1.04 (0.66–1.64) 1.15 (0.72–1.82) 1.30 (0.81–2.09)	0.659	*N = 1,473* 0.03 (0.01–0.04) 0.03 (0.02–0.04) 0.03 (0.02–0.05) 0.04 (0.02–0.06)	**0.006**	*N=369* 2.04 (1.19–2.89) 2.19 (1.90–2.47) 2.17 (1.76–2.58) NA	0.955
Any antibiotic use in past 14 days: - Yes - No	*N = 587* 2.4 97.6	*N = 119* 4.2 95.8	1.80 (0.63–5.08) Reference	0.293	*N = 1,173* 0.10 (0.06–0.14) 0.04 (0.03–0.05)	**0.048**	2.32 (1.94–2.69) 2.10 (1.83–2.38)	0.363
Current urogenital symptom: - Yes - No	*N = 588* 42.7 57.3	*N = 120* 42.5 57.5	0.99 (0.67–1.48) Reference	0.970	*N = 1,044* 0.03 (0.02–0.05) 0.04 (0.03–0.05)	0.655	2.33 (1.71–2.95) 2.15 (1.91–2.39)	0.481
Current unusual vaginal discharge: - Yes - No	*N = 588* 17.7 82.3	*N = 120* 12.5 87.5	0.66 (0.37–1.19) Reference	0.153	*N = 1,044* 0.02 (0.00–0.04) 0.04 (0.03–0.05)	0.371	2.65 (1.21–4.08) 2.16 (1.94–2.38)	0.386
Tested HIV-positive:^f^ - Yes - No	40.0 60.0	39.8 60.2	0.99 (0.72–1.36) Reference	0.953	*N = 1,641* 0.03 (0.02–0.03) 0.03 (0.02–0.04)	** <0.001**	*N = 126* 1.91 (−22.4–26.2) 1.84 (1.45–2.22)	0.983
Nugent score categories:^f^ - 0–3 - 4–6 - 7–10	*N = 477* 38.2 16.6 45.3	*N = 96* 17.7 40.6 41.7	Reference 5.29 (2.82–9.90) 1.98 (1.09–3.62)	** <0.001**	*N = 893* 0.01 (0.01–0.02) 0.06 (0.04–0.09) 0.02 (0.01–0.03)	** <0.001**	*N = 364* 1.61 (1.29–1.94) 2.78 (2.16–3.40) 2.25 (1.92–2.59)	**0.001**
Yeasts by microscopy:^f^ - Yes - No	*N = 477* 8.0 92.0	*N = 98* 8.2 91.8	1.03 (0.46–2.27) Reference	0.948	*N = 905* 0.02 (0.00–0.04) 0.03 (0.03–0.04)	0.846	*N = 374* 2.21 (1.33–3.08) 2.18 (1.94–2.41)	0.912
*Trichomonas vaginalis* by culture/NAAT:^f^ - Yes - No	*N = 935* 7.7 92.3	*N = 194* 11.3 88.7	1.53 (0.93–2.54) Reference	0.108	*N = 1,462* 0.02 (0.01–0.04) 0.03 (0.03–0.04)	0.806	*N = 373* 2.92 (1.89-3.95) 2.15 (1.92-2.37)	0.133
*Chlamydia trachomatis* by NAAT:^f^ - Yes - No	*N = 936* 6.5 93.5	*N = 194* 4.6 94.4	0.70 (0.34–1.43) Reference	0.307	*N = 1,195* 0.02 (0.00-0.05) 0.03 (0.02-0.03)	0.930	*N = 126* 2.74 (1.81-3.67) 1.60 (1.19-2.01)	**0.019**
*Neisseria gonorrhoeae* by NAAT:^f^ - Yes - No	*N = 936* 2.2 97.8	*N = 194* 2.6 97.4	1.15 (0.43–3.10) Reference	0.781	*N = 1,195* 0.01 (0–0.03) 0.03 (0.02–0.03)	0.650	*N = 126* 2.78 (1.67–3.89) 1.68 (1.28–2.08)	**0.043**
*Mycoplasma genitalium* by NAAT:^f^ - Yes - No	*N = 369* 8.9 91.1	*N = 76* 6.6 93.4	0.72 (0.27–1.90) Reference	0.489	*N = 445* 0.02 (0.00–0.03) 0.03 (0.02–0.04)	0.896	NA	NA
Herpes simplex virus type 2 by serology:^f^ - Yes - No	*N = 503* 87.1 12.9	*N = 102* 91.2 8.8	1.53 (0.74–3.19) Reference	0.232	*N = 627* 0.02 (0.01–0.03) 0.02 (0.00–0.04)	**0.054**	*N = 87* 2.03 (1.33–2.74) 1.67 (1.02–2.33)	0.410
Active syphilis by serology:^f^ - Yes - No	*N = 502* 2.8 97.2	*N = 102* 2.0 98.0	0.70 (0.16–3.12) Reference	0.622	*N = 668* 0.01 (−0.01–0.03) 0.02 (0.02–0.03)	0.293	*N = 126* 1.87 (−1.33–5.07) 1.84 (1.45–2.22)	0.918
High-risk HPV by PCR:^f^ - Yes - No	*N = 821* 52.5 47.5	*N = 170* 50.6 49.4	0.93 (0.67–1.29) Reference	0.650	*N = 1,415* 0.03 (0.02–0.03) 0.03 (0.02–0.04)	0.107	NA	NA

*CI, confidence interval; HPV, human papilloma virus; IUD, intrauterine device; NA, not applicable; NAAT, nucleic acid amplification test; ND, not determinable; OR, odds ratio; PCR, polymerase chain reaction*.

a *Refer to the footnotes of Table 2 for other details regarding the independent variables tested in these logistic regression models*.

b *Logistic regression analysis with total pathobionts relative abundance (≥1 vs. <1%) as the outcome. All models contained the outcome and one independent variable*.

c *By Kruskall–Wallis test, comparing mean pathobionts relative abundances or estimated concentrations between independent variable categories. For age, Spearman's rank correlation was used, correlating age as a continuous variable with pathobionts relative abundances or estimated concentrations as continuous variables*.

d *VMB study samples collected at the screening and Month 6 visits in all randomization groups, and at the Month 1 and Month 2 visits in the no-intervention group, and all HARP and HELIUS samples, were considered not influenced by interventions*.

e *Menses data are only available for follow-up visits in the Rwanda VMB study*.

f *Includes samples from all study visits at which this outcome was tested (excluding invalid results, if applicable)*.

## Discussion

Seventeen percent of this highly diverse group of women from Africa and Europe had a VMB containing at least 1% pathobionts, and 5.2% had a VMB containing at least 20% pathobionts. *Streptococcus* was most common (54% of the pathobionts sequencing reads), but *Staphylococcus, Enterococcus, Escherichia/Shigella, Haemophilus*, and *Campylobacter* were also detected as non-minority genera in all three studies. Mean relative abundances and estimated concentrations of pathobionts were much lower than those of lactobacilli and BV-anaerobes, but the pathogenic potential may be higher, and these levels may therefore be clinically relevant.

The meta-analysis confirmed that the VMB of many women contains both lactobacilli and BV-anaerobes, but that the BV-anaerobes concentration is low in “healthy” women with lactobacilli-domination. Our relative abundance, estimated concentration, and correlation data may be best explained by the following hypothesis. BV-anaerobes are present or frequently introduced into the vagina of most women, and may start to expand in response to a trigger, such as recent sex or menses (Jespers et al., 2017). When the BV-anaerobes concentration increases, the lactobacilli concentration does not seem to decline much initially, but instead, the total bacterial concentration increases. The lactobacilli relative abundance therefore does decline. We cannot test this hypothesis directly because our analyses were cross-sectional, but the strong negative correlation between lactobacilli and BV-anaerobes relative abundances (ρ = −0.9234) but not estimated concentrations (*r* = 0.0031), and the higher overall bacterial load of the anaerobic dysbiotic VMB types (means 6.11–6.87 log_10_ cells/μl) compared to the lactobacilli-dominated VMB types (means 5.13–5.83 log_10_ cells/μl) fit this hypothesis.

By contrast, a much smaller proportion of women in our study carried pathobionts in their VMB (17% if a 1% relative abundance is used as a cut-off). Our relative abundance, estimated concentration, and correlation data may be best explained by the following hypothesis. Pathobionts are occasionally introduced into the vagina from the gut, urinary tract, and perineum, or from the male partner external genitalia, but are usually cleared or remain at low levels. If they do persist and expand, lactobacilli decline somewhat, and BV-anaerobes expand alongside the pathobionts. As before, we cannot test this hypothesis directly, but the modest positive correlations between estimated concentrations of pathobionts and BV-anaerobes, the declines in estimated concentration/relative abundance of lactobacilli with increasing pathobionts level, and an overall bacterial load of the ≥20% pathobionts VMB type (5.85 log_10_ cells/μl) that is similar to that of the lactobacilli-dominated VMB types (means 5.13–5.83 log_10_ cells/μl) fit this hypothesis. Pathobionts also correlated positively with the “other bacteria” group, which contains non-pathogenic skin bacteria such as *Corynebacterium*. The pathobionts and non-pathogenic skin bacteria may have been introduced into the vagina from the woman's perineum or the skin of the external genitalia of her male partner, but specimen contamination via the hands of specimen handlers cannot be ruled out.

Gram stain Nugent scoring is the current gold standard for BV diagnosis (Nugent et al., 1991). In this method, Gram stained slides are viewed under a microscopy, and three bacterial morphotypes are scored: Gram-positive rods (presumed to be lactobacilli), small Gram-variable rods (presumed to be *G. vaginalis*), and curved Gram-variable rods (presumed to be *Mobiluncus*). A Nugent score of 0–3 is considered BV-negative, 4–6 intermediate microbiota, and 7–10 BV-positive. In this meta-analysis, the likelihood of detection, mean relative abundance, and mean estimated concentration of pathobionts were consistently highest for Nugent score category 4–6, followed by 7–10, and 0–3. These findings also fit the above-mentioned hypotheses, and provide a partial explanation for what a Nugent score of 4–6 signifies. A Nugent score of 4–6 should, however, not be used to diagnose pathobionts presence because another significant proportion of these samples likely contain lactobacilli plus BV-anaerobes.

Positive associations between pathobionts detection and/or levels and young age, non-Dutch origin, hormonal contraceptive use, smoking, antibiotic use in the 14 days prior to sampling, HIV status, and the presence of STI pathogens were found in at least one study. All of these factors are also risk factors for anaerobic dysbiosis, except for hormonal contraceptive use. Hormonal contraception, and especially methods containing estrogen, protects women from anaerobic dysbiosis (van de Wijgert et al., 2013). Authors have hypothesized that estrogen increases vaginal glycogen, which is converted into lactic acid by lactobacilli. This keeps BV-anaerobes at bay but perhaps not pathobionts. Streptococci, for example, can tolerate low vaginal pH very well (Shabayek and Spellerberg, 2017). Sexual risk-taking is an important proven risk factor for anaerobic dysbiosis (van de Wijgert et al., 2014), but associations with pathobionts detection and/or levels were inconsistent in this meta-analysis. This could be due to the fact that women in the two African cohorts were recruited based on sexual risk or HIV-status, and women in the Dutch cohort were not, thereby introducing collinearity between study/ethnic group and sexual risk. Associations between pathobionts detection/levels and unusual vaginal discharge reporting were also inconsistent between studies. In our experience, vaginal symptom-reporting rarely correlates well with the actual presence of a vaginal infection or vaginal dysbiosis (Verwijs et al., 2019b). None of the women in the three studies had severe symptoms, such as those associated with desquamative inflammatory vaginitis, and we could therefore not test the association between pathobionts levels and such symptoms.

A limitation of our study is that each of the three studies used slightly different sequencing-related laboratory and initial data processing methods (Table 1). However, we took this into account by stratifying most analyses by study. Another limitation is that we did not detect and quantify individual pathobiont species and genera by quantitative PCR. Past *S. agalactiae* prevalence studies using selective culture have shown average rectovaginal detection rates of 22% in sub-Saharan Africa and 19% in Europe (Kwatra et al., 2016), and a recent study using quantitative PCR found a vaginal detection rate of 20% in Kenyan women and 23% in South African women (Cools et al., 2016). Detection of vaginal *Streptococcus* was lower in our meta-analysis when a relative abundance cut-off of 1% was used (12.7% of all women at baseline; about a quarter of those were *S. agalactiae*). It is currently not known how rectovaginal and vaginal selective cultures, PCR, and sequencing results relate to one another, but it is possible that pathobionts are not only under-detected in sequencing studies due to the bioinformatics used, but also due to DNA extraction, amplification, and other biases. For example, the detection rate in the HARP study was especially low, which may have been due to the fact that we did not use bead-beating during DNA extraction in that study (Gill et al., 2016). Third, some of the standard diagnostic tests that we used are known to have lower sensitivity than NAAT-based tests (e.g., culture for vulvovaginal candidiasis and *T. vaginalis*), and not all women in all three studies were screened for all STI pathogens.

We also report some limitations related to our statistical analyses. Correlating variables derived from relative abundance data is problematic because they are not independent (Knight et al., 2018); estimated concentrations of these same variables are independent and did indeed provide new insights as described above. However, we only had estimated concentration data for the Rwanda VMB study. Furthermore, our analyses were cross-sectional, and some of them had limited statistical power. Our findings are therefore hypothesis-generating and the hypotheses should be tested in well-powered longitudinal studies that assess the VMB quantitatively. We did not exclude all women who had recently used antibiotics, but reported antibiotic use in the last 2 weeks was rare. A strength of our study is the inclusion of women and samples from three world regions and multiple ethnic groups, and with different behaviors and STI pathogen exposures. The variability in VMB compositions that we observed reflects this wide variety of study participants.

## Conclusion

While substantial presence of pathobionts in the VMB was less common than anaerobic dysbiosis, the pathogenic potential of pathobionts is higher than that of BV-anaerobes, and modest levels could therefore be clinically relevant. The most frequently used VMB types, and analyses limited to relative abundance, are inadequate. We recommend that future etiologic and intervention studies quantify the most common vaginal pathobiont genera, as well as lactobacilli and BV-anaerobes. Furthermore, the various detection and quantification methods (culture, PCR, and sequencing) should be rigorously compared to one another to facilitate interpretation of clinical study results.

## Data Availability Statement

The three studies included in this meta-analyses were governed by different institutions and ethics committees. Data availability therefore differs for each study. The three original publications, which are referenced in this publication, include data availability statements for each respective study. Additional data unique to this publication are provided in Supplementary Material 1.

## Ethics Statement

The Rwanda VMB study was sponsored by the University of Liverpool, approved by the Rwanda National Ethics Committee and the University of Liverpool Research Ethics Subcommittee for Physical Interventions, and registered on ClinicalTrials.gov (NCT02459665). The South African portion of the HARP study was approved by the ethics committees of the University of Witwatersrand in Johannesburg and the London School of Hygiene and Tropical Medicine. The HELIUS study was approved by the Medical Ethics Committee of the Academic Medical Center in Amsterdam (protocol number: 10/100; amendment 10/100# 10.17.1729; NL32251.018.10). The patients/participants provided their written informed consent to participate in this study.

## Author Contributions

JW was the Principal Investigator of all three VMB sequencing studies. PM was the Principal Investigator of the HARP parent study. MV, AG, HB, and CV contributed to the VMB sequencing laboratory work, processed the initial sequencing data, and compiled the metadata datasets. MV and JW wrote the data analysis plan. MV compiled the combined dataset and conducted the analyses presented in this paper. JW and MV wrote the manuscript. All authors commented on and approved the manuscript.

### Conflict of Interest

The authors declare that the research was conducted in the absence of any commercial or financial relationships that could be construed as a potential conflict of interest.

## Supplementary Material

Supplementary Material 1 List of taxa identified in the three studies and classification into bacterial groups.
