# Supplementary material for: Pathobionts in the Vaginal Microbiota: Individual Participant Data Meta-Analysis of Three Sequencing Studies
Source: Front Cell Infect Microbiol. 2020 Apr 15;10:129. doi: 10.3389/fcimb.2020.00129 (PMC7174631; doi:10.3389/fcimb.2020.00129)
Supplement: Supplementary file 2 [file Data_Sheet_2.docx]

**Supplement 2**

Manuscript title: Pathobiont Presence in the Vaginal Microbiota: Individual Participant Data Meta-analysis of Three Sequencing Studies.

Manuscript authors: Janneke H.H.M. van de Wijgert, Marijn C. Verwijs, Alexandra C. Gill, Hanneke Borgdorff, Charlotte van der Veer, and Philippe Mayaud on behalf of the Rwanda VMB, HARP, and HELIUS study groups.

This document contains the supplementary tables and figures belonging to the above-mentioned manuscript. The manuscript concerns an individual participant data meta-analysis of three vaginal microbiota (VMB) sequencing studies. The references for the three main VMB manuscripts of the three original studies are listed below. These manuscripts contain detailed information on study design, study implementation, laboratory methods, sequencing data processing, and data analysis, and these are therefore not repeated here. The details that are most relevant for the interpretation of the data presented in this manuscript are listed in Table 1 of the manuscript.

**References for the three original VMB sequencing studies:**

1. Rwanda VMB Study: Janneke H.H.M. van de Wijgert, Marijn C. Verwijs1, Stephen K. Agaba, Christina Bronowski, Lambert Mwambarangwe, Mireille Uwineza, Elke Lievens, Adrien Nivoliez, Jacques Ravel and Alistair C. Darby. Intermittent lactobacilli-containing vaginal probiotic or metronidazole use to prevent bacterial vaginosis recurrence: safety and preliminary efficacy by microscopy and sequencing. *MedRxiv* 2019. doi:10.1101/19001156.
2. HARP VMB Study: Janneke H.H.M. van de Wijgert, A. Christina Gill, Admire Chikandiwa, Marijn C. Verwijs, Helen A. Kelly, Tanvier Omar, Sinead Delany-Moretlwe, Michel Segondy, Suzanna Francis, Alistair C. Darby, Philippe Mayaud, for the HARP Study Group. Human papillomavirus infection and cervical dysplasia in HIV-positive women: potential role of the vaginal microbiota. *AIDS* 2019. doi:10.1097/QAD.0000000000002381.
3. HELIUS VMB Study: Hanneke Borgdorff, Charlotte van der Veer, Robin van Houdt, Catharina J. Alberts, Henry J. de Vries, Sylvia M. Bruisten, Marieke B. Snijder, Maria Prins, Suzanne E. Geerlings, Maarten F. Schim van der Loeff, Janneke H. H. M. van de Wijgert. The association between ethnicity and vaginal microbiota composition in Amsterdam, the Netherlands. *PLOS ONE* 2017; 12, e0181135. doi:10.1371/journal.pone.0181135.

**Figure S1: Heatmap of all samples from all three studies (N=2,044)**


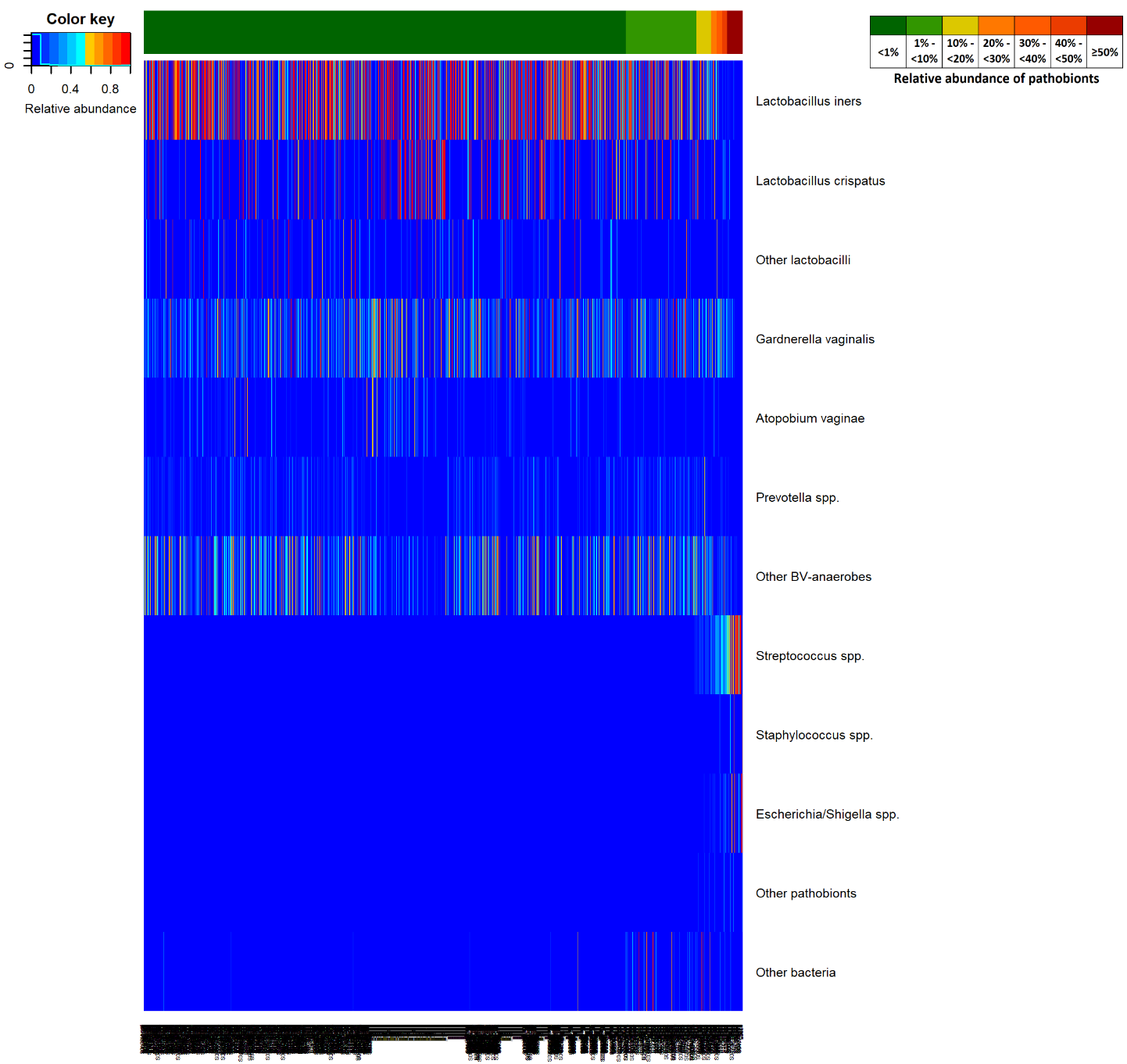


Abbreviation: *BV* bacterial vaginosis.

This heatmap includes all samples from all three studies (N=2,044) by increasing relative abundance of total pathobionts per sample (from left to right).

**Table S1: Description of all samples with pathobiont presence ≥20%**

|  | **Description (percentages are relative abundances)** | **Study** | **N** |
| --- | --- | --- | --- |
| **Samples dominated by pathobionts (≥50% relative abundance)^1^; *N=53*** | | | |
| *Streptococcus-*dominated | Between 53-98% streptococci and no other pathobionts.   - 13/34: 50-98% *Streptococcus agalactiae/pyogenes;* one also includes 11% *S. anginosus/ milleri* and 18% *S. dentisani/infantis*, another 10% *S. anginosus*. - 1/34: 37% *S. agalactiae/pyogenes* and 39% *S. dentisani/infantis*. - 1/34: 97% *S. dysgalactiae/ pyogenes*. - 13/34: 41-75% *S. dentisani/infantis*; most also including *Streptococcus* genus (up to 24%). - 1/34: 61% *S. anginosus/milleri*, while another 69% *S. anginosus*. - 2/34: 72% and 93% *S. equinus/infantarius/lutetiensis,* respectively*.* - 1/34: 71% *Streptococcus* genus. | Total | 33 |
|  |  | VMB | 15 |
|  |  | HARP | 10 |
|  |  | HELIUS | 8 |
| *Staphylococcus*-dominated | 82% *Staphylococcus* genus with 12.5% *Lactobacillus iners*. | Total | 1 |
|  |  | HELIUS | 1 |
| *Escherichia/*  *Shigella*-dominated | Between 64-94% *Escherichia*/*Shigella* genus, 2/5 in combination with BV-associated anaerobes, and 1/5 also contains 5% of the pathobiont *Morganella morganii*. | Total | 5 |
|  |  | VMB | 3 |
|  |  | HARP | 1 |
|  |  | HELIUS | 1 |
| Mixed patho-bionts with >10% *Streptococcus* | - 1/11: 73% *S. agalactiae/pyogenes* with 10% *S. argenteus/aureus* and *S. aureus/capitis.* - 1/11: 51% *Streptococcus* genus with 8% *S. anginosus/milleri,* 10% *Enterococcus durans/faecalis/faecium*, and 7% other *Enterococcus* species. - 1/11: 47% *S. dentisani/infantis*, 14% *Streptococcus* genus, and 9% *Haemophilus* genus. - 1/11: 46% *S. agalactiae*/*pyogenes* and 10% *Escherichia*/*Shigella*. - 1/11: 34% *S. anginosus/milleri*, 12% *S. dentisani/infantis*, and 42% *Escherichia/Shigella*. - 1/11: 32% *S. anginosus/milleri*, 11% *S. dentisani/infantis*, 15% *S. aureus/ capitis*, and 27% *S. epidermidis/haemolyticus/hominis*. - 1/11: 27% *S. anginosus*, 9% *Streptococcus* genus and 16% *Escherichia/Shigella*. - 1/11: 55% *E. faecalis* with 14% *S. anginosus* and 12% *S. agalactiae.* - 1/11: 21% *Streptococcus* genus, 21% *E. durans/faecalis/faecium* and 10% *Neisseria gonorrhoeae/meningitidis*. - 1/11: 13% *S. dentisani/infantis*, 32% *S. aureus/devriesei/epidermidis/haemolyticus*, 29% *S. haemolyticus*, and 15% *S. epidermidis/haemolyticus*. - 1/11: 32% *Raoultella* genus with 13% *S. agalactiae*, 6% *Proteus* genus, 5% *Staphylococcus* genus and 37% *L. iners*. | Total | 11 |
|  |  | VMB | 6 |
|  |  | HARP | 2 |
|  |  | HELIUS | 3 |
| Mixed patho-bionts without *Streptococcus* | - 1/3: 36% *S. aureus/capitis* and 19% *E. durans/faecalis*. - 1/3: 86% *Escherichia*/*Shigella* and 12% *Haemophilus* genus. - 1/3: 76% *Escherichia*/*Shigella* and 20% *Haemophilus* genus*.* | Total | 3 |
|  |  | VMB | 2 |
|  |  | HARP | 1 |
| **Samples with substantial presence of pathobionts (20-50% relative abundance)^1^; *N=54*** | | | |
| Substantial *Streptococcus* | Between 16-49% *Streptococcus* but species vary greatly: Mostly *S. agalactiae* or *S. agalactiae/pyogenes* in 15/42, *S. dentisani/infantis* in 9/42, *S. anginosus* or *S. anginosus/milleri* in 8/42, *S. alactolyticus/equinus* in 2/42, *S. equinus/infantarius* in 2/42, *Streptococcus* genus in 1/42, and approximately equal distribution of multiple species in 5/42 (2/5 *Streptococcus* genus and *S. dentisani/infantis*, 1/5 *Streptococcus* genus and *S. equinus/infantarius*, 1/5 *S. agalactiae* and *S. anginosus,* and 1/5 *S. anginosus/milleri* and *S. alactolyticus/equinus*). 17/42 samples also contain >5% *L. iners* (5-76%), 5/42 >5% *L. crispatus* (10-32%), and 8/42 >5% other *Lactobacillus* species (6-61%). 24/42 samples also contain >5% *Gardnerella* (6-70%) and 10/42 >5% *Prevotella* (5-31%). *Atopobium* and *Sneathia* were also common. | Total | 42 |
|  |  | VMB | 18 |
|  |  | HARP | 16 |
|  |  | HELIUS | 8 |
| Substantial *Staphylococcus* | - 1/2: 19% *S. aureus/devriesei/epidermidis/haemolyticus*, 11% *S. epidermidis/ haemolyticus*, 41% *Mycoplasma hominis*, and 8% *Corynebacteria*. - 1/2: 15% *S. aureus/devriesei*, 13% *S. haemolyticus*, 7% *S. epidermidis/haemolyticus*, 21% *Sneathia*, and 16% *Granulicatella elegans*. | Total | 2 |
|  |  | VMB | 1 |
|  |  | HARP | 1 |
| Substantial *Enterococcus* | - 1/2: 17% *E. azikeevi/durans/faecalis*, 4% *Escherichia/Shigella*, 67% *Prevotella* species*,* and 9% other BV-anaerobes. - 1/2: 44% *E. faecalis* with 2% *Escherichia/ Shigella* and 44% *G. vaginalis*. | Total | 2 |
|  |  | VMB | 1 |
|  |  | HELIUS | 1 |
| Substantial *Escherichia/ Shigella* | Between 38-45% *Escherichia/Shigella*. Additional bacteria: 1/3 21% *L. crispatus* and 16% *L. iners*; 1/3 15% *L. iners*, 17% *Gardnerella*, and 11% *Prevotella* species; and 1/3 22% *Fusobacterium equinum/gonidiaformans* and 21% *Bacteroides fragilis*. | Total | 3 |
|  |  | VMB | 2 |
|  |  | HELIUS | 1 |
| Substantial *Haemophilus* | 21% *Haemophilus* genus and 75% *G. vaginalis*. | Total | 1 |
|  |  | HELIUS | 1 |
| Mixed pathobionts | - 1/4: 16% *Escherichia/Shigella*, 7% *Streptococcus dentisani/infantis*, and 76% *L. iners*. - 1/4: 23% *Escherichia/Shigella*, 9% *Streptococcus* genus, 21% *Bifidobacteria*, 24% *G. vaginalis* and 8% *Prevotella* species. - 1/4: 25% *Enterococcus durans/faecalis*, 20% *S. epidermidis/haemolyticus*, 50% *L. iners*. - 1/4: 13% *Enterococcus azikeevi/durans/faecalis*, 13% *Escherichia/Shigella*, and 71% *L. crispatus*. | Total | 4 |
|  |  | VMB | 2 |
|  |  | HELIUS | 1 |
|  |  | HARP | 1 |

Abbreviations: *BV* bacterial vaginosis. N are numbers of samples.

1. All taxa with relative abundance of 5% or higher are listed. The ‘substantial *Escherichia/Shigella*’sample contained 4% *Streptococcus* but the other samples contained <1% *Streptococcus*.

*Campylobacter* was detected at 1-3.4% in a total of 13 samples, but none of these had pathobionts ≥20% (5 had VMB type LA and 8 had VMB type BV_noGV).

**Table S2A:** **Correlation matrix of relative abundances, all samples from all studies (N=2,044)**

|  | **Total lactobacilli** | **Total BV-**  **anaerobes** | **Total other**  **bacteria** | **Total pathobionts** | ***Strepto-coccus*** | ***Staphylo-coccus*** | ***Entero-coccus*** | ***Escherichia*/**  ***Shigella*** | ***Campylo-bacter*** | ***Haemo-philus*** |
| --- | --- | --- | --- | --- | --- | --- | --- | --- | --- | --- |
| **Total lactobacilli** | 1 | **-0.9234** | **-0.2235** | **-0.2076** | **-0.1671** | **0.0927** | 0.0036 | **-0.0713** | **-0.1370** | **-0.0936** |
| **Total BV-anaerobes** | **-0.9234** | 1 | **0.1118** | 0.0160 | 0.0021 | **-0.2034** | **-0.0994** | **-0.0625** | **0.1271** | -0.0191 |
| **Total other bacteria** | **-0.2235** | **0.1118** | 1 | **0.3831** | **0.3182** | **0.3552** | **0.1881** | **0.1753** | **0.2078** | **0.0875** |
| **Total pathobionts** | **-0.2076** | 0.016 | **0.3831** | 1 | **0.8142** | **0.4891** | **0.2811** | **0.3718** | **0.2307** | **0.2727** |
| ***Streptococcus*** | **-0.1671** | 0.0021 | **0.3182** | **0.8142** | 1 | 0.3505 | 0.2382 | **0.2570** | **0.0527** | **0.2486** |
| ***Staphylococcus*** | **0.0927** | **-0.2034** | **0.3552** | **0.4891** | **0.3505** | 1 | 0.2800 | **0.2324** | **0.1037** | **0.1262** |
| ***Enterococcus*** | 0.0036 | **-0.0994** | **0.1881** | **0.2811** | **0.2382** | **0.2800** | 1 | **0.3001** | **0.0579** | **0.0648** |
| ***Escherichia*/*Shigella*** | **-0.0713** | **-0.0625** | **0.1753** | **0.3718** | **0.2570** | **0.2324** | **0.3001** | 1 | 0.0382 | **0.1587** |
| ***Campylobacter*** | **-0.1370** | **0.1271** | **0.2078** | **0.2307** | **0.0527** | **0.1037** | **0.0579** | 0.0382 | 1 | -0.0200 |
| ***Haemophilus*** | **-0.0936** | -0.0191 | **0.0875** | **0.2727** | **0.2486** | **0.1262** | **0.0648** | **0.1587** | -0.0200 | 1 |

Abbreviations: *BV* bacterial vaginosis; *VMB* vaginal microbiota.

Cells contain Spearman’s rank correlation coefficients. Correlation coefficients shown in bold were statistically significant at p<0.05.

This correlation matrix was also done for each individual study but results were similar and did not generate any new insights.

**Table S2B: Correlation matrix of estimated concentrations, all samples Rwanda VMB study (N=379)**

|  | **Total lactobacilli** | **Total BV-**  **anaerobes** | **Total other**  **bacteria** | **Total pathobionts** | ***Strepto-coccus*** | ***Staphylo-coccus*** | ***Entero-coccus*** | ***Escherichia*/**  ***Shigella*** | ***Campylo-bacter*** | ***Haemo-philus*** |
| --- | --- | --- | --- | --- | --- | --- | --- | --- | --- | --- |
| **Total lactobacilli** | 1 | 0.0031 | 0.0437 | 0.0436 | 0.0677 | -0.0042 | -0.0466 | -0.0012 | -0.0978 | -0.0744 |
| **Total BV-anaerobes** | 0.0031 | 1 | **0.3530** | **0.1938** | **0.1525** | **-0.1337** | **-0.1369** | -0.0722 | **0.1568** | -0.0344 |
| **Total other bacteria** | 0.0437 | **0.3530** | 1 | **0.3388** | **0.3033** | **0.1959** | **0.1025** | **0.1404** | **0.1036** | 0.0376 |
| **Total pathobionts** | 0.0436 | **0.1938** | **0.3388** | 1 | **0.8177** | **0.3912** | **0.2513** | **0.4205** | **0.1470** | **0.1922** |
| ***Streptococcus*** | 0.0677 | **0.1525** | **0.3033** | **0.8177** | 1 | **0.3262** | **0.2054** | **0.2837** | -0.0757 | **0.2294** |
| ***Staphylococcus*** | -0.0042 | **-0.1337** | **0.1959** | **0.3912** | **0.3262** | 1 | **0.2743** | **0.3335** | -0.0757 | **0.1723** |
| ***Enterococcus*** | -0.0466 | **-0.1369** | **0.1025** | **0.2513** | **0.2054** | **0.2743** | 1 | **0.3907** | -0.0415 | **0.1158** |
| ***Escherichia*/*Shigella*** | -0.0012 | -0.0722 | **0.1404** | **0.4205** | **0.2837** | **0.3335** | **0.3907** | 1 | -0.0734 | 0.0795 |
| ***Campylobacter*** | -0.0978 | **0.1568** | **0.1036** | **0.1470** | -0.0757 | -0.0757 | -0.0415 | -0.0734 | 1 | -0.0282 |
| ***Haemophilus*** | -0.0744 | -0.0344 | 0.0376 | **0.1922** | **0.2294** | **0.1723** | **0.1158** | 0.0795 | -0.0282 | 1 |

Abbreviations: *BV* bacterial vaginosis; *VMB* vaginal microbiota.

Cells contain Pearson’s correlation coefficients. Correlation coefficients shown in bold were statistically significant at p<0.05.

Estimated concentrations are only available for the Rwanda VMB study.

**Table S2C: Correlation matrix of relative abundances, all samples not influenced by interventions (N=1,781)^1^**

|  | **Total lactobacilli** | **Total BV-**  **anaerobes** | **Total other**  **bacteria** | **Total pathobionts** | ***Strepto-coccus*** | ***Staphylo-coccus*** | ***Entero-coccus*** | ***Escherichia*/**  ***Shigella*** | ***Campylo-bacter*** | ***Haemo-philus*** |
| --- | --- | --- | --- | --- | --- | --- | --- | --- | --- | --- |
| **Total lactobacilli** | 1 | **-0.9250** | **-0.1991** | **-0.1851** | **-0.1397** | **0.1280** | 0.0176 | **-0.0768** | **-0.1305** | **-0.0889** |
| **Total BV-anaerobes** | **-0.9250** | 1 | **0.0865** | -0.0012 | -0.0190 | **-0.2324** | **-0.1097** | **-0.0500** | **0.1151** | -0.0195 |
| **Total other bacteria** | **-0.1991** | **0.0865** | 1 | **0.3894** | **0.3129** | **0.3549** | **0.1741** | **0.1749** | **0.2166** | **0.0786** |
| **Total pathobionts** | **-0.1851** | -0.0012 | **0.3894** | 1 | **0.8090** | **0.4979** | **0.2678** | **0.3357** | **0.2586** | **0.2666** |
| ***Streptococcus*** | **-0.1397** | -0.0190 | **0.3129** | **0.8090** | 1 | **0.3494** | **0.2328** | **0.2299** | **0.0661** | **0.2385** |
| ***Staphylococcus*** | **0.1280** | **-0.2324** | **0.3549** | **0.4979** | **0.3494** | 1 | **0.2887** | **0.2335** | **0.1101** | **0.1065** |
| ***Enterococcus*** | 0.0176 | **-0.1097** | **0.1741** | **0.2678** | **0.2328** | **0.2887** | 1 | **0.2797** | **0.0715** | **0.0787** |
| ***Escherichia*/*Shigella*** | **-0.0768** | **-0.0500** | **0.1749** | **0.3357** | **0.2299** | **0.2335** | **0.2797** | 1 | **0.0733** | **0.1594** |
| ***Campylobacter*** | **-0.1305** | **0.1151** | **0.2166** | **0.2586** | **0.0661** | **0.1101** | **0.0715** | **0.0733** | 1 | -0.0157 |
| ***Haemophilus*** | **-0.0889** | -0.0195 | **0.0786** | **0.2666** | **0.2385** | **0.1065** | **0.0787** | **0.1594** | -0.0157 | 1 |

Abbreviations: *BV* bacterial vaginosis; *VMB* vaginal microbiota.

Cells contain Spearman’s rank correlation coefficients. Correlation coefficients shown in bold were statistically significant at p<0.05.

This correlation matrix was also done for each individual study but results were similar and did not generate any new insights.

^1^ Excluding Enrolment and Day 7 samples from all four randomization groups and Month 1 and Month 2 samples of women randomized to the three intervention groups.

**Table S2D: Correlation matrix of estimated concentrations, Rwanda VMB study samples not influenced by interventions (N=158)^1^**

|  | **Total lactobacilli** | **Total BV-**  **anaerobes** | **Total other**  **bacteria** | **Total pathobionts** | ***Strepto-coccus*** | ***Staphylo-coccus*** | ***Entero-coccus*** | ***Escherichia*/**  ***Shigella*** | ***Campylo-bacter*** | ***Haemophilus*** |
| --- | --- | --- | --- | --- | --- | --- | --- | --- | --- | --- |
| **Total lactobacilli** | 1 | 0.0739 | 0.0367 | -0.0132 | 0.0458 | -0.0105 | -0.0942 | -0.1499 | -0.1336 | -0.0748 |
| **Total BV-anaerobes** | 0.0739 | 1 | **0.2810** | 0.1009 | 0.0777 | **-0.2749** | -0.1386 | -0.0616 | 0.1301 | -0.1062 |
| **Total other bacteria** | 0.0367 | **0.2810** | 1 | **0.2652** | **0.1877** | 0.0818 | -0.0004 | 0.0624 | **0.1578** | -0.0527 |
| **Total pathobionts** | -0.0132 | 0.1009 | **0.2652** | 1 | **0.8135** | **0.3701** | **0.2119** | **0.3784** | **0.1804** | **0.2238** |
| ***Streptococcus*** | 0.0458 | 0.0777 | **0.1877** | **0.8135** | 1 | **0.3206** | **0.2261** | **0.1590** | -0.0211 | **0.2613** |
| ***Staphylococcus*** | -0.0105 | **-0.2749** | 0.0818 | **0.3701** | **0.3206** | 1 | **0.2958** | **0.3187** | -0.0840 | **0.1709** |
| ***Enterococcus*** | -0.0942 | -0.1386 | -0.0004 | **0.2119** | **0.2261** | **0.2958** | 1 | **0.3415** | -0.0374 | **0.4804** |
| ***Escherichia*/*Shigella*** | -0.1499 | -0.0616 | 0.0624 | **0.3784** | **0.1590** | **0.3187** | **0.3415** | 1 | -0.0696 | 0.0773 |
| ***Campylobacter*** | -0.1336 | 0.1301 | **0.1578** | **0.1804** | -0.0211 | -0.0840 | -0.0374 | -0.0696 | 1 | -0.0347 |
| ***Haemophilus*** | -0.0748 | -0.1062 | -0.0527 | **0.2238** | **0.2613** | **0.1709** | **0.4804** | 0.0773 | -0.0347 | 1 |

Abbreviations: *BV* bacterial vaginosis; *VMB* vaginal microbiota.

Cells contain Pearson’s correlation coefficients. Correlation coefficients shown in bold were statistically significant at p<0.05.

Estimated concentrations are only available for the Rwanda VMB study.

^1^ Excluding Enrolment and Day 7 samples from all four randomization groups and Month 1 and Month 2 samples of women randomized to the three intervention groups.

**Table S3A: Correlates of pathobionts detection, relative abundance, and concentration – Rwanda VMB study only**

|  | **Rwanda VMB study screening samples** | | | | **All Rwanda VMB study samples** | | | |
| --- | --- | --- | --- | --- | --- | --- | --- | --- |
| **Independent variables^1^** | **<1%**  **(% of N=136)** | **≥1%**  **(% of N=26)** | **OR (95% CI)^2^** | ***P*^2^** | **Mean relative abundance (95% CI); N=629** | ***P*^3^** | **Mean concentration log_10_ cells/µl (95% CI); N=379** | ***P*^3^** |
| Potentially influenced by interventions:^4^   - Yes - No | NA^4^ | NA^4^ | NA^4^ | NA^4^ | 0.07 (0.04-0.09)  0.05 (0.03-0.07) | 0.216 | 2.28 (1.99-2.57)  2.04 (1.69-2.38) | 0.318 |
| Age categories:  - 18 – 24  - 25 – 29  - 30 – 34  - 35 – 44 | 14.7  24.3  36.7  24.3 | 19.2  34.6  19.2  26.9 | Reference  1.09 (0.32-3.72)  0.40 (0.10-1.53)  0.85 (0.24-3.04) | 0.328 | 0.19 (0.12-0.26)  0.05 (0.03-0.07)  0.02 (0.01-0.03)  0.05 (0.03-0.06) | **<0.001** | 3.09 (2.56-3.62)  2.29 (1.80-2.78)  1.77 (1.37-2.16)  1.95 (1.57-2.32) | **0.001** |
| Contraceptive use:  - None or condom use only  - Any oral contraception  - Progestin-only injectable  - Progestin-only implant  - Any IUD (copper or hormonal)  - Contraceptive ring  - NA (currently pregnant) | 44.6  9.2  29.2  15.4  1.5  0  6.0 | 23.1  15.4  26.9  30.8  3.9  0  0 | Reference  3.22 (0.79-13.19)  1.78 (0.56-5.71)  3.87 (1.20-12.51)  4.83 (0.38-61.49)  NA  ND | 0.163 | *N=366*  0.02 (0-0.03)  0.02 (0-0.04)  0.07 (0.03-0.11)  0.04 (0.01-0.07)  0.01 (-0.01-0.03)  NA  0 (0-0) | **<0.001** | *N = 252*  1.53 (1.11-1.95)  2.89 (1.93-3.86)  2.37 (1.86-2.88)  2.58 (2.00-3.17)  1.55 (0.05-3.04)  NA  1.27 (-4.21-6.75) | **0.014** |
| Any hormonal contraception or pregnant:   - Yes - No | 55.9  44.1 | 73.1  26.9 | 2.14 (0.85-5.43)  Reference | 0.096 | *N=366*  0.05 (0.03-0.07)  0.02 (0-0.03) | **<0.001** | *N = 252*  2.50 (2.15-2.85)  1.53 (1.13-1.93) | **<0.001** |
| Current smoker:   - Yes - No | NA | NA | NA | NA | NA | NA | NA | NA |
| Sample taken:^5^  - During or within 7 days after menses  - Not during or within 7 days after menses | NA | NA | NA | NA | *N=462*  0.05 (0.02-0.09)  0.08 (0.06-0.10) | 0.236 | *N = 310*  2.12 (1.63-2.62)  2.25 (1.97-2.53) | 0.606 |
| Any type of vaginal cleansing:   - Yes - No | 15.7  84.3 | 17.3  82.7 | 1.13 (0.58-2.20)  Reference | 0.732 | *N=330*  0.06 (0.01-0.10)  0.05 (0.03-0.07) | 0.727 | *N = 310*  2.47 (1.82-3.11)  2.18 (1.92-2.44) | 0.417 |
| Number of sex partners prior to sampling:   - None - One - Two or more | 0  7.4  92.7 | 0  3.9  96.2 | NA  Reference  1.98 (0.24-16.20) | 0.486 | *N=362*  0 (-0.01-0.01)  0.02 (0-0.05)  0.04 (0.02-0.05) | 0.309 | *N=249*  1.81 (-21.19-24.82)  1.82 (1.21-2.43)  2.17 (1.87-2.48) | 0.571 |
| Frequency of condom use:  - Never  - Inconsistent  - Consistent  - NA (no sexual partner) | 5.9  72.8  21.3  0 | 3.9  61.5  34.6  0 | Reference  1.29 (0.15-11.04)  2.48 (0.27-22.62)  NA | 0.357 | *N=484*  0.02 (0-0.04)  0.04 (0.02-0.05)  0.05 (0.03-0.08)  NA | 0.836 | *N=369*  2.04 (1.19-2.89)  2.19 (1.90-2.47)  2.17 (1.76-2.58)  NA | 0.955 |
| Any antibiotic use in past 14 days:   - Yes - No | 0  100 | 0  100 | NA | NA | 0.11 (0.07-0.15)  0.04 (0.03-0.05) | **0.043** | 2.32 (1.94-2.69)  2.10 (1.83-2.38) | 0.363 |
| Current urogenital symptom:   - Yes - No | 43.4  56.6 | 53.9  46.1 | 1.52 (0.66-3.54) | 0.327 | *N=498*  0.03 (0.01-0.04)  0.04 (0.03-0.06) | 0.585 | 2.33 (1.71-2.95)  2.15 (1.91-2.39) | 0.481 |
| Current unusual vaginal discharge:   - Yes - No | 11.8  88.2 | 19.2  80.8 | 1.79 (0.59-5.40) | 0.322 | *N=498*  0.05 (0-0.10)  0.04 (0.03-0.05) | 0.696 | 2.65 (1.21-4.08)  2.16 (1.94-2.38) | 0.386 |

|  | **Rwanda VMB study screening samples** | | | | **All Rwanda VMB study samples** | | | |
| --- | --- | --- | --- | --- | --- | --- | --- | --- |
| **Independent variables^1^** | **<1%**  **(% of N=136)** | **≥1%**  **(% of N=26)** | **OR (95% CI)^2^** | ***P*^2^** | **Mean relative abundance (95% CI); N=626** | ***P*^3^** | **Mean concentration log_10_ cells/µl (95% CI); N=379** | ***P*^3^** |
| Tested HIV-positive:^6^   - Yes - No | 10.3  89.7 | 7.7  92.3 | 0.73 (0.15-3.40)  Reference | 0.675 | *N=226*  0 (0-0.01)  0.02 (0.01-0.03) | 0.204 | *N = 126*  1.91 (-22.4-26.2)^6^  1.84 (1.45-2.22) | 0.983^6^ |
| Nugent score categories:^6^  - 0-3  - 4-6  - 7-10 | 41.4  11.2  47.4 | 13.6  27.3  59.1 | Reference  7.38 (1.62-33.61)  3.78 (1.02-14.07) | **0.017** | *N=458*  0.02 (0.01-0.03)  0.03 (0.01-0.05)  0.03 (0.02-0.04) | **<0.001** | *N = 364*  1.61 (1.29-1.94)  2.78 (2.16-3.40)  2.25 (1.92-2.59) | **0.001** |
| Yeasts by microscopy:^6^   - Yes - No | 9.5  90.5 | 12.5  87.5 | 1.36 (0.35-5.31) | 0.662 | *N=470*  0.01 (0-0.03)  0.04 (0.03-0.05) | 0.943 | *N = 374*  2.21 (1.33-3.08)  2.18 (1.94-2.41) | 0.912 |
| *Trichomonas vaginalis* by culture/NAAT:^6^   - Yes - No | 7.9  92.1 | 33.3  66.7 | 5.83 (1.97-17.31) | **0.002** | *N=471*  0.04 (0.01-0.07)  0.04 (0.03-0.06) | **0.038** | *N = 377*  2.92 (1.89-3.95)  2.15 (1.92-2.37) | 0.133 |
| *Chlamydia trachomatis* by NAAT:^6^   - Yes - No | 21.7  78.3 | 20.8  79.2 | 0.95 (0.32-2.79) | 0.922 | *N=204*  0.02 (0-0.04)  0.02 (0.01-0.03) | 0.226 | *N = 126*  2.74 (1.81-3.67)  1.60 (1.19-2.01) | **0.019** |
| *Neisseria gonorrhoeae* by NAAT:^6^   - Yes - No | 12.2  87.8 | 16.7  83.3 | 1.44 (0.43-4.84) | 0.562 | *N=204*  0.02 (0-0.04)  0.02 (0.01-0.03) | 0.193 | *N = 126*  2.78 (1.67-3.89)  1.68 (1.28-2.08) | **0.043** |
| *Mycoplasma genitalium* by NAAT:^6^   - Yes - No | NA | NA | NA | NA |  |  | NA | NA |
| Herpes simplex virus type 2 by serology:^6^   - Yes - No | 66.9  33.1 | 69.2  30.8 | 1.11 (0.45-2.75) | 0.817 | *N=184*  0.01 (0.01-0.02)  0.01 (0-0.02) | 0.771 | *N = 87*  2.03 (1.33-2.74)  1.67 (1.02-2.33) | 0.410 |
| Active syphilis by serology:^6^   - Yes - No | 8.1  91.9 | 7.7  92.3 | 0.95 (0.20-4.55) | 0.945 | *N=226*  0.01 (-0.01-0.03)  0.02 (0.01-0.03) | 0.580 | *N = 126*  1.87 (-1.33-5.07)  1.84 (1.45-2.22) | 0.918 |
| High-risk HPV by PCR:^6^   - Yes - No | NA | NA | NA | NA |  |  | NA | NA |

Abbreviations: *HPV* human papilloma virus; *CI* confidence interval; *IUD* intrauterine device; *NA* not applicable; *NAAT* nucleic acid amplification test; *ND* not determinable; *OR* odds ratio; *PCR* polymerase chain reaction.

1. Refer to the footnotes of Table 2 for other details regarding the independent variables tested in these logistic regression models.
2. Logistic regression analysis with total pathobionts relative abundance (≥1% versus <1%) as the outcome. All models contained the outcome and one independent variable.
3. By Kruskall Wallis test, comparing mean pathobionts relative abundances or concentrations between independent variable categories. For age, Spearman’s rank correlation was used, correlating age as a continuous variable with pathobionts relative abundances or concentrations as continuous variables.
4. Rwanda VMB study samples collected at the screening and Month 6 visits in all randomization groups, and at the Month 1 and Month 2 visits in the no-intervention group were considered not influenced by interventions.
5. Menses data are only available for follow-up visits in the Rwanda VMB study.
6. Includes samples from all study visits at which this outcome was tested (excluding invalid results, if applicable).

**Table S3B: Correlates of pathobionts detection and relative abundance - HARP study only**

|  | **HARP baseline samples** | | | | **All HARP samples** | |
| --- | --- | --- | --- | --- | --- | --- |
| **Independent variables^1^** | **<1%**  **(% of369)** | **≥1%**  **(% of 76)** | **OR (95% CI)^2^** | ***P*^2^** | **Mean relative abundance (95% CI); N=869** | ***P*^3^** |
| Age categories:  - 25 – 29  - 30 – 34  - 35 – 44  - 45 – 50 | 19.0  30.1  44.2  6.8 | 21.1  32.9  36.8  9.2 | Reference  0.99 (0.49-1.97)  0.75 (0.38-1.48)  1.23 (0.45-3.33) | 0.658 | 0.05 (0.03-0.08)  0.02 (0.01-0.03)  0.02 (0.01-0.03)  0.04 (0-0.07) | 0.217 |
| Contraceptive use:  - None or condom use only  - Any oral contraception  - Progestin-only injectable  - Progestin-only implant  - Any IUD (copper or hormonal)  - Contraceptive ring  - NA (currently pregnant) | 74.3  4.6  19.8  1.4  0  0  0 | 79.0  7.9  11.8  1.3  0  0  0 | Reference  1.61 (0.61-4.26)  0.56 (0.27-1.19)  0.91 (0.10-7.96)  NA  NA  NA | 0.288 | *N=859*  0.02 (0.02-0.03)  0.07 (0.01-0.13)  0.04 (0.02-0.06)  0 (0-0.01)  NA  NA  NA | 0.621 |
| Any hormonal contraception or pregnant:   - Yes - No | 25.6  74.4 | 21.1  78.9 | 0.77 (0.42-1.40)  Reference | 0.382 | *N=859*  0.04 (0.02-0.06)  0.02 (0.02-0.03) | 0.731 |
| Current smoker:   - Yes - No | 4.9  95.1 | 9.2  90.8 | 1.98 (0.80-4.92)  Reference | 0.161 | 0.02 (0-0.04)  0.03 (0.02-0.04) | 0.948 |
| Any type of vaginal cleansing:   - Yes - No | 38.2  61.8 | 39.5  60.5 | 1.05 (0.64-1.75)  Reference | 0.837 | 0.03 (0.01-0.05)  0.02 (0.01-0.04) | **0.010** |
| Number of sex partners prior to sampling:   - None - One - Two or more | 18.2  77.5  4.3 | 10.7  77.3  12.0 | Reference  1.70 (0.77-3.73)  4.71 (1.57-14.12) | **0.023** | *N=868*  0.02 (0-0.04)  0.03 (0.02-0.03)  0.08 (0.02-0.14) | **0.012** |
| Frequency of condom use:  - Never  - Inconsistent  - Consistent  - NA (no sexual partner) | 5.2  26.6  49.1  19.2 | 2.6  39.5  47.4  10.5 | Reference  2.91 (0.64-13.21)  1.89 (0.42-8.47)  1.07 (0.21-5.46) | 0.059 | *N=445*  0 (0-0.01)  0.04 (0.02-0.07)  0.02 (0.01-0.04)  0.01 (0-0.02) | 0.540 |
| Any antibiotic use in past 14 days | NA | NA | NA | NA | NA | NA |
| Current urogenital symptom | NA | NA | NA | NA | NA | NA |
| Current unusual vaginal discharge | NA | NA | NA | NA | NA | NA |
| Tested HIV-positive:^4^ | 100 | 100 | NA | NA | NA | NA |
| Nugent score categories:^4^  - 0-3  - 4-6  - 7-10 | 37.1  18.3  44.6 | 18.9  44.6  36.5 | Reference  4.79 (2.40-9.55)  1.61 (0.81-3.18) | **<0.001** | *N=435*  0.01 (0-0.01)  0.08 (0.04-0.12)  0.01 (0-0.02) | **<0.001** |
| Yeasts by microscopy:^4^   - Yes - No | 7.5  92.5 | 6.8  93.2 | 0.90 (0.33-2.41)  Reference | 0.827 | *N=435*  0.03 (-0.01-0.07)  0.03 (0.02-0.04) | 0.842 |
| *Trichomonas vaginalis* by culture/NAAT:^4^   - Yes - No | 15.7  84.3 | 15.8  84.2 | - 1. (0.51-1.98)   Reference | 0.988 | *N=445*  0.02 (0-0.03)  0.03 (0.02-0.04) | 0.553 |
| *Chlamydia trachomatis* by NAAT:^4^   - Yes - No | 5.2  94.8 | 4.0  96.0 | 0.76 (0.22-2.63)  Reference | 0.651 | *N=445*  0.05 (-0.02-0.12)  0.02 (0.01-0.03) | 0.873 |
| *Neisseria gonorrhoeae* by NAAT:^4^   - Yes - No | 1.9  98.1 | 1.3  98.7 | 0.69 (0.08-5.69)  Reference | 0.718 | *N=445*  0 (0-0.01)  0.03 (0.02-0.04) | 0.341 |
| *Mycoplasma genitalium* by NAAT:^4^   - Yes - No | 8.9  91.1 | 6.6  93.4 | 0.72 (0.27-1.90)  Reference | 0.489 | *N=445*  0.02 (0-0.03)  0.03 (0.02-0.04) | 0.896 |
| Herpes simplex virus type 2 by serology:^4^   - Yes - No | 94.6  5.4 | 98.7  1.3 | 4.32 (0.57-32.71)  Reference | 0.077 | *N=443*  0.02 (0.01-0.03)  0.04 (-0.05-0.13) | **0.013** |
| Active syphilis by serology:^4^   - Yes - No | 3.0  97.0 | 0  100 | NA | NA | *N=442*  0 (0-0)  0.03 (0.02-0.04) | 0.353 |
| High-risk HPV by PCR:^4^   - Yes - No | 78.6  21.4 | 84.2  15.8 | 1.45 (0.75-2.82)  Reference | 0.257 | 0.03 (0.02-0.04)  0.03 (0.01-0.04) | **0.003** |

Abbreviations: *HPV* human papilloma virus; *CI* confidence interval; *IUD* intrauterine device; *NA* not applicable; *NAAT* nucleic acid amplification test; *OR* odds ratio; *PCR* polymerase chain reaction.

1. Refer to the footnotes of Table 2 for other details regarding the independent variables tested in these logistic regression models.
2. Logistic regression analysis with total pathobionts relative abundance (≥1% versus <1%) as the outcome. All models contained the outcome and one independent variable.
3. By Kruskall Wallis test, comparing mean pathobionts relative abundances/concentrations between independent variable categories. Spearman’s rank correlation was used to correlate age as a continuous variable with pathobionts relative abundance or concentration.
4. Includes samples from all study visits at which this outcome was tested (excluding invalid results, if applicable).

**Table S3C: Correlates of pathobionts detection and relative abundance - HELIUS study only**

|  | **All HELIUS samples** | | | | **All HELIUS samples** | |
| --- | --- | --- | --- | --- | --- | --- |
| **Independent variables^1^** | **<1%**  **(% of 452)** | **≥1%**  **(% of 94)** | **OR (95% CI)^2^** | ***P*^2^** | **Mean relative abundance (95% CI); N=546** | ***P*^3^** |
| Age categories:  - 18 – 24  - 25 – 29  - 30 – 34 | 37.4  31.6  31.0 | 39.4  30.9  29.8 | Reference  0.93 (0.54-1.58)  0.91 (0.53-1.57) | 0.937 | 0.04 (0.02-0.06)  0.03 (0.01-0.04)  0.03 (0.01-0.05) | 0.973 |
| Ethnicity:  - sub-Saharan African origin  - Turkish, Moroccan, South-Asian origin  - Dutch origin | 35.8  44.9  19.3 | 22.3  64.9  12.8 | 0.94 (0.44-2.00)  2.18 (1.12-4.25)  Reference | **0.002** | 0.03 (0.01-0.05)  0.04 (0.03-0.06)  0.01 (0-0.03) | **<0.001** |
| Contraceptive use:  - None or condom use only  - Any oral contraception  - Progestin-only injectable  - Progestin-only implant  - Any IUD (copper or hormonal)  - Contraceptive ring  - NA (currently pregnant) | 48.6  37.2  0.5  0.9  11.6  1.3  0 | 57.5  29.8  0  2.1  8.5  2.1  0 | Reference  0.68 (0.41-1.11)  NA  2.02 (0.36-11.31)  0.62 (0.28-1.38)  1.35 (0.26-6.85)  NA | 0.363 | *N=543*  0.04 (0.02-0.06)  0.02 (0.01-0.04)  NA  0 (0-0)  0.01 (0-0.03)  0.02 (-0.02-0.05) NA | 0.095 |
| Any hormonal contraception or pregnant:   - Yes - No | 45.1  54.9 | 37.2  62.8 | 0.72 (0.45-1.17)  Reference | 0.179 | *N=483*  0.02 (0.01-0.04)  0.04 (0.01-0.04) | 0.053 |
| Current smoker:   - Yes - No | 24.2  75.8 | 17.0  83.0 | 0.64 (0.36-1.15)  Reference | 0.121 | *N=544*  0.01 (0-0.03)  0.04 (0.02-0.05) | **0.004** |
| Any type of vaginal cleansing:   - Yes - No | 25.9  74.1 | 21.3  78.7 | 0.77 (0.17-0.29)  Reference | 0.336 | *N=545*  0.01 (0-0.02)  0.04 (0.03-0.05) | 0.533 |
| Number of sex partners prior to sampling:   - None - One - Two or more | 30.6  55.4  14.0 | 44.7  45.7  9.6 | Reference  0.57 (0.35-0.91)  0.47 (0.22-1.02) | **0.031** | *N=545*  0.05 (0.02-0.07)  0.03 (0.01-0.04)  0.01 (0-0.03) | **<0.001** |
| Frequency of condom use:  - Never  - Inconsistent  - Consistent  - NA (no sexual partner) | 37.6  21.6  10.2  30.7 | 35.1  10.6  9.6  44.7 | Reference  0.53 (0.25-1.12)  1.00 (0.45-2.24)  1.56 (0.94-2.59) | **0.020** | *N=544*  0.03 (0.01-0.05)  0.01 (0-0.02)  0.02 (0-0.05)  0.05 (0.02-0.07) | **<0.001** |
| Any antibiotic use in past 14 days:   - Yes - No | 3.1  96.9 | 5.4  94.6 | 1.77 (0.62-5.05)  Reference | 0.305 | *N=544*  0.04 (-0.01-0.10)  0.03 (0.02-0.04) | **0.042** |
| Current urogenital symptom:   - Yes - No | 42.5  57.5 | 39.4  60.6 | 0.88 (0.56-1.38)  Reference | 0.577 | 0.04 (0.02-0.06)  0.03 (0.02-0.04) | 0.134 |
| Current unusual vaginal discharge:   - Yes - No | 19.5  80.5 | 10.6  89.4 | 0.49 (0.25-0.99)  Reference | **0.033** | 0.02 (0-0.04)  0.04 (0.02-0.06) | **0.011** |
| Tested HIV-positive | NA | NA | NA | NA | NA | NA |
| Nugent score categories | NA | NA | NA | NA | NA | NA |
| Yeasts by microscopy | NA | NA | NA | NA | NA | NA |
| *Trichomonas vaginalis* by culture/NAAT:   - Yes - No | 1.1  98.9 | 2.1  97.9 | 1.94 (0.37-10.17)  Reference | 0.455 | 0.01 (0-0.03)  0.03 (0.02-0.04) | 0.185 |
| *Chlamydia trachomatis* by NAAT:   - Yes - No | 3.8  96.2 | 1.1  98.9 | 0.28 (0.04-2.09)  Reference | 0.131 | 0 (0-0.01)  0.03 (0.02-0.04) | 0.817 |
| *Neisseria gonorrhoeae* by NAAT:   - Yes - No | 0  100 | 0  100 | NA | NA | NA | NA |
| *Mycoplasma genitalium* by NAAT | NA | NA | NA | NA |  |  |
| Herpes simplex virus type 2 by serology | NA | NA | NA | NA |  |  |
| Active syphilis by serology | NA | NA | NA | NA |  |  |
| High-risk HPV by PCR:   - Yes - No | 31.2  68.8 | 23.4  76.6 | 0.67 (0.40-1.13)  Reference | 0.126 | 0.02 (0.01-0.04)  0.04 (0.02-0.05) | 0.057 |

Abbreviations: *HPV* human papilloma virus; *CI* confidence interval; *IUD* intrauterine device; *NA* not applicable; *NAAT* nucleic acid amplification test; *OR* odds ratio; *PCR* polymerase chain reaction.

1. Refer to the footnotes of Table 2 for other details regarding the independent variables tested in these logistic regression models.
2. Logistic regression analysis with total pathobionts relative abundance (≥1% versus <1%) as the outcome. All models contained the outcome and one independent variable.
3. By Kruskall Wallis test, comparing mean pathobionts relative abundances/concentrations between independent variable categories. Spearman’s rank correlation was used to correlate age as a continuous variable with pathobionts relative abundance or concentration.
4. Includes samples from all study visits at which this outcome was tested (excluding invalid results, if applicable).
